# Cobalt–Carbon Nanoparticles with Silica Support for Uptake of Cationic and Anionic Dyes from Polluted Water

**DOI:** 10.3390/molecules26247489

**Published:** 2021-12-10

**Authors:** Hassan H. Hammud, Ranjith Kumar Karnati, Nusaybah Alotaibi, Syed Ghazanfar Hussain, Thirumurugan Prakasam

**Affiliations:** 1 Department of Chemistry, College of Science, King Faisal University, P.O. Box 400, Al-Ahsa 31982, Saudi Arabia; Rkarnati@kfu.edu.sa (R.K.K.); nalotaibi@kfu.edu.sa (N.A.); 2 Department of Physics, College of Science, King Faisal University, P.O. Box 400, Al-Ahsa 31982, Saudi Arabia; sghazanfar@kfu.edu.sa; 3 Chemistry Program, New York University Abu Dhabi (NYUAD), Abu Dhabi, United Arab Emirates; tp42@nyu.edu

**Keywords:** pyrolysis, cobalt, nanocarbon, silica, surface analysis, adsorption, water treatment

## Abstract

Silica-supported hierarchical graphitic carbon sheltering cobalt nanoparticles Co-HGC@SiO_2_ (1) were prepared by pyrolysis at 850 °C of [Co(phen)(H_2_O)_4_]SO_4_·2H_2_O complex with silica in the presence of pyrene as a carbon source under nitrogen atmosphere. Nanocomposites (2) and (3) were obtained by acid treatment of (1) with HCl and HF acid, respectively. The nanocomposites showed rough hierarchical carbon microstructures over silica support decorated with irregular cobalt nanospheres and nanorods 50 to 200 nm in diameter. The nanoparticles consist of graphitic shells and cobalt cores. SEM, EDAX and TEM elemental mapping indicate a noticeable loss of cobalt in the case of (2) and loss of cobalt and silica in the case of (3) with an increase in porosity. Nanocomposite (3) showed the highest BET surface area 217.5 m^2^g^−1^. Raman spectrum shows defect D-band and graphitic G-band as expected in carbon nanostructures. PXRD reveals the presence of cobalt(0) nanoparticles. XPS indicates the presence of Co(II) oxides and the successful doping of nitrogen in the nanocomposites. Moreover, TEM elemental mapping provides information about the abundance of Si, Co, C, N and S elements in zones. Nanocomposite (1) showed maximum uptake capacity of 192.3 and 224.5 mg/g for crystal violet CV and methyl orange MO dyes, respectively. Nanocomposite (2) showed a capacity of 94.1 and 225.5 mg/g for CV and MO dyes, respectively. Nanocomposite (4) obtained after treatment of (1) with crystal violet proved successful adsorption of CV. Co-HGC (5) prepared without addition of silica has a capacity for CV equal to 192 mg/g, while it is 769.2 mg/g with MO. Electrostatics and π–π interactions of graphite and cobalt species in the nanocomposites with aromatic rings of cationic and anionic dyes are responsible for the adsorption. Yan et al. was the best model to describe column kinetics. The thomas column adsorption model showed that the maximum uptake capacity of (1) was 44.42 mg/g for CV and 32.62 mg/g for MO. for a column packed with 0.5 gm of (1) and dye concentration of 100 mg/L at a flow rate of 1 mL/min. The column was recycled three times with no noticeable clogging or degradation of nanocomposites. Thus, Co-HGC@SiO_2_ adsorbents can be used efficiently to treat water contaminated with cationic and anionic dyes.

## 1. Introduction 

Textiles, paper, leather, and other industry materials use substantial amounts of reactive dyes. For instance, textile industry alone produces 7,000,000 tons of dyes annually [1] and accounts for about 1–20% of dye effluents [2]. Because of their chemical stability, the discharged dyes resist physical, chemical and biological degradation [3]. 

One of the harmful dyes that is extensively used in many industries is methylene orange (MO). It is an anionic aromatic azo dye that is extensively used in research laboratories, textiles and other industries [4]. It poses serious harmful effects to humans, including carcinogenic and mutagenic, and is toxic to aquatic life [5]. Because of its resistance to natural degradation, MO removal from wastewater is a key challenge. Besides MO, crystal violet dye (CV) is extensively used in medication, biological stain, mutagenic, bacteriostatic agent and fungal growth inhibitors. It is also used as a dye in the textile and paint industries [6]. CV is a cationic aromatic triarylmethane dye and it causes harmful effects to humans, such as severe kidney failure [7], skin irritation, respiratory problems and cancer [8,9]. It is a biohazardous material that resists degradation in the environment [6]. 

Removing toxic dyes from wastewater has become a serious environmental concern. Many effluent treatment techniques have been developed in order to remove toxic dyes from water, such as coagulation, flocculation [10], filtration [11], biodegradation [12] and electrochemical decolorization [13]. Among dye removal methods, the adsorption technique is an effective alternative due to several factors, such as simplicity, cost-efficiency and feasibility. The most widely used adsorbents for dye removal with high adsorption capacity are carbon-based materials [14,15,16]. For example, Ma et al. reported alkali-activated multiwalled carbon nanotubes used in the removal of both cationic Methylene Blue (MB) and anionic dyes (MO) [17]. Teng et al. prepared carbon nanofibers using a sol-gel/electrospinning method and used them in the adsorption of dyes including MO [18]. Liu et al., prepared multiwalled carbon nanotubes using hydrothermal carbonization of glucose and employed them as adsorbents for removal of cationic dyes MB and CV [19]. 

Remarkable applications of metal–carbon nanostructures have considerably accelerated the development of preparative methodology with desirable physical and chemical properties. Initially, metal cations were deposited on carbonaceous materials, then reduced using chemical or physical reduction methods [20,21]. This process suffers from heterogeneous dispersion and agglomeration of the metallic particles. Therefore, different methods have been explored to overcome the agglomeration issue and obtain well-distributed particles on the carbon matrix. These methods are thermal decomposition and arc discharge of organic/metal complex mixture under optimized conditions [22]. Guerret-Piecourt and co-workers prepared cobalt nanorods of 200 nm length encapsulated in carbon nanotubes using arc discharge [23]. In addition, chemical vapor deposition (CVD) has been used to prepare metal nanoparticles filled in carbon nanotubes. For instance, Chatterjee et al. prepared carbon nanotubes 10–50 nm in length filled with cobalt nanoparticles using thermal decomposition of Co(NO_3_)_2_ and urea at 600 °C. Then, the residue was used as a catalyst to grow carbon nanotubes (CNTs) by CVD of turpentine oil [24]. Laser ablation has also been used to prepare metal carbon nanocomposites. Kwong et al. prepared cobalt nanoparticles of 5–70 nm size encapsulated in a graphitic shell with the thickness of a few nanometers using laser ablation in toluene [25]. 

In addition, large-scale pyrolytic synthesis of cobalt–carbon nanostructures with new properties using transition metal complexes precursors appears to be an alternative method for preparing carbon nanostructures. It was found that the affinity between cobalt complexes and alkynes increases the reactivity of organometallic compounds [26]. The appropriate precursor can be designed using a supramolecular building approach [26]. Dosa and co-workers reported that heating dehydrobenzoannulene (DBA) and Co_2_(CO)_8_ at 800 °C for six hours led to the formation of multi-walled CNTs, graphitic and amorphous carbon, where cobalt metal appeared as dark dots on the sidewalls [27]. Highly crystalline and graphitic carbon nanopipes and nanocapsules were obtained by pyrolysis of Fe(II) and Co(II) gluconates at pyrolysis temperature of 900 °C or 1000 °C [28]. El Hamaoui et al., reported that the thermal decomposition of polyphenylene dendrimer/cobalt complexes produced CNTs, uniform carbon/cobalt nanorods, and carbon/cobalt nanospheres [29]. Laskoski et al., reported the synthesis of graphitic carbon tubes and onions by pyrolysis of Co-carbonyl dehydro[n]anulenes [30]. Pyrolysis of polyphenylene–cobalt complexes was conducted by El Hamaoui et al. to obtain a variety of carbon nanostructures. They observed that the type and the structure of CNTs depend on the shape and composition of the precursors [31]. In addition, they obtained CNTs and bamboo-like CNTs by pyrolysis of the cobalt complex with hexa-*peri*-hexabenzocoronene [32]. 

Cobalt–carbon nanostructures have been used as an adsorbent for both cationic and anionic dyes for water treatment application. For instance, Hammud et al., prepared cobalt–carbon nanostructures using solid-state pyrolysis of *cis*-dichlorobis(1,10-phenanthroline-N,N′)-cobalt(II) complex and anthracene and used it in the adsorption of malachite green dye with a capacity of 492 mg/g [33]. Tang et al., prepared cobalt nanoparticles embedded in mesoporous carbon using an infusing/calcination method and used it for the adsorption of rhodamine B with a capacity of 468 mg/g [34]. Ye et al. prepared cobalt nanoparticles deposited on carbon microrods using solid-state pyrolysis of cobalt 1,3,5-benzenetricarboxylate MOF [Co_3_(BTC)_2_(H_2_O)_12_] and employed it to remove methyl blue, acid fuchsin, malachite green, rhodamine B, methyl orange and methylene blue [35]. 

Many dye adsorbents have some disadvantages that limit their use as effective adsorbents, such as high cost, complicated preparation method, incapability of regeneration, high energy input and low chemical and physical stability. 

Therefore, there is an urgent need for low-cost and large-scale production of an efficient regenerable adsorbent for both cationic and anionic aromatic dyes. The adsorbent should have high chemical and physical stability that can be used in continuous adsorption system on industrial scale. Crystal violet CV is a triarylmethane cationic dye containing three dimethyl amine groups. When CV is dissolved in water at pH greater than 2, the dye has a blue-violet colour, [7,36]. Methyl orange MO is a pH indicator is red in acidic, orange in neutral and yellow in basic solution. It is a diarylazo sulfonate anionic dye containing one dimethyl amine group [4,36].

We present a novel simple method to prepare cobalt nanocarbon with silica support to be used as adsorbents for removal of anionic and cationic aromatic dyes. Only very few closely related adsorbents are reported in the literature, but without silica matrix [34,35]. Our method is considered to be economically suitable, since in addition to the easily prepared cobalt phenanthroline complex, cheap materials pyrene and abundant silica were used as starting materials in (1:1:1). The yield of adsorbent increases when using silica as starting materials. The cobalt graphitic carbons form on silica surface an adsorptive coat that can be efficient and selective for uptake of aromatic dyes thru π-π interactions. Cobalt nanoparticles are expected to enhance adsorption thru binding with electron donors group present in the dyes. Therefore, porous silica-supported hierarchical graphitic carbon sheltering cobalt nanoparticles Co-HGC@SiO_2_ can be produced at large scale. In addition, silica support makes the nanocomposite robust and regenerable that can be easily packed into a column, reducing its clogging for better use in water treatment in the industry.

## 2. Experimental

Cobalt(II) sulfate heptahydrate (99%, SDFCL), 1,10-phenanthroline (+99%, Acros Organics, Fairrun, NJ, USA), ethanol (ACS grade, Scharlau, Germany), sodium hydroxide (pure, Acros Organic, Geel, Belgium), anthracene (99.0%), methylene orange (99.5%), and crystal violet dye (99%) were Merck brand. Remaining all other reagents and starting materials were purchased from Sigma-Aldrich and used without further purification.

### 2.1. Preparation of Samples

Preparation of cobalt complex (precursor): [Co(phen)(H_2_O)_4_]SO_4_·2H_2_O was prepared by mixing CoSO_4_·7H_2_O (4.0 mmol), and 1,10-phenanthroline (4.0 mmol) in 30 mL water. After 3h stirring, pink powder precipitate was collected by filtration, washed with water and dried in an oven at 60 °C to give (2.4 mmol) (60% yield) [37]. 

**Scheme 1:**
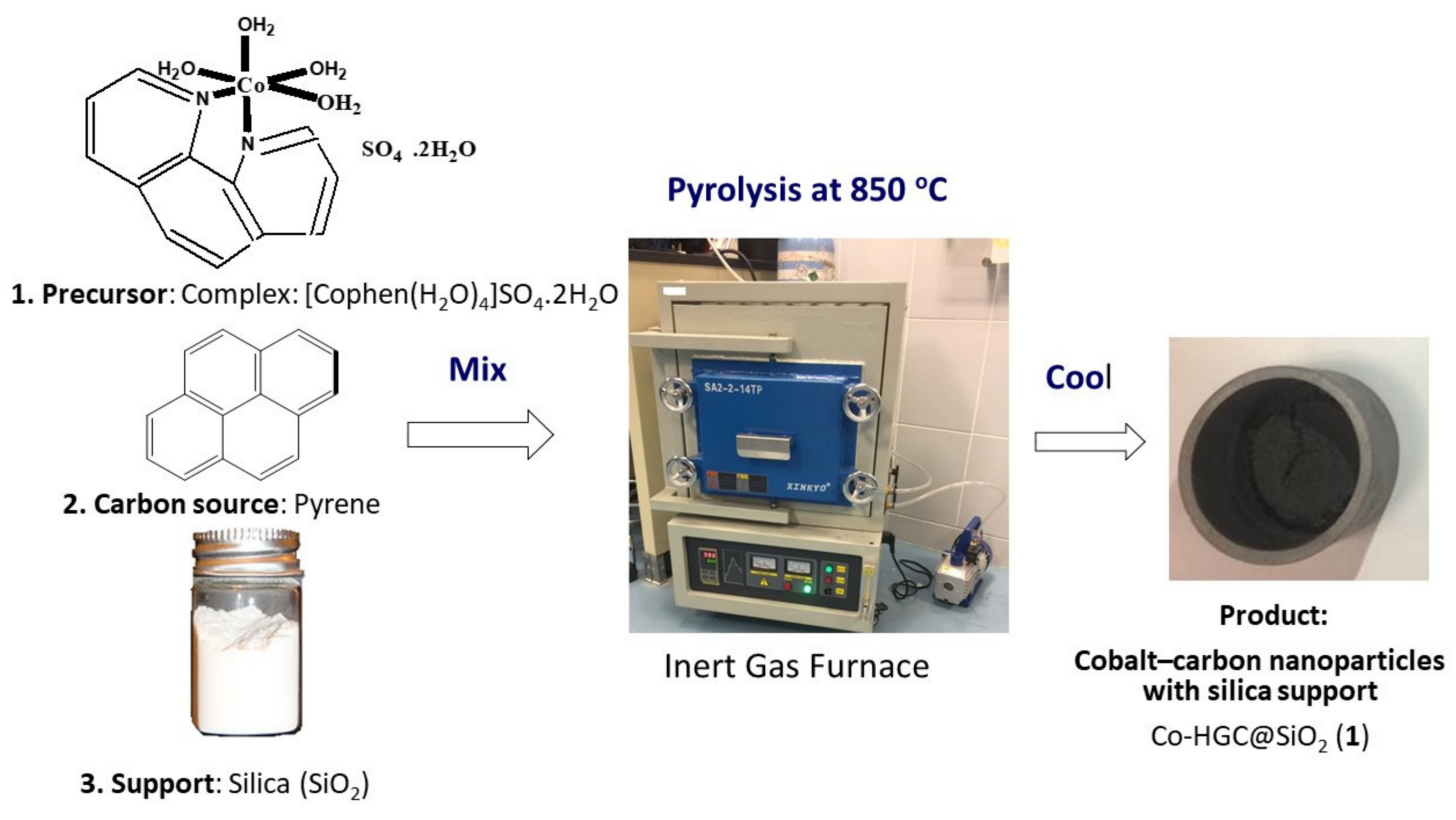
Graphic illustration of preparation of Co-HGC@SiO_2_ (**1**).

Preparation of Co-HGC@SiO_2_ (1): [Co(phen)(H_2_O)_4_]SO_4_·2H_2_O (1.5 g), anthracene (1.5 g), and silica gel-60 (1.5 g) were mixed and grounded well together in a porcelain crucible and placed inside a furnace under low pressure of nitrogen. Then, the reaction mixture was heated first at 300 °C for 2 h and next at 850 °C for 15 h (Final Stage), to ensure complete graphitization. After slow cooling, 1.86 g of (1) was obtained as a fine dark black powder, Scheme 1.

**Scheme 2:**
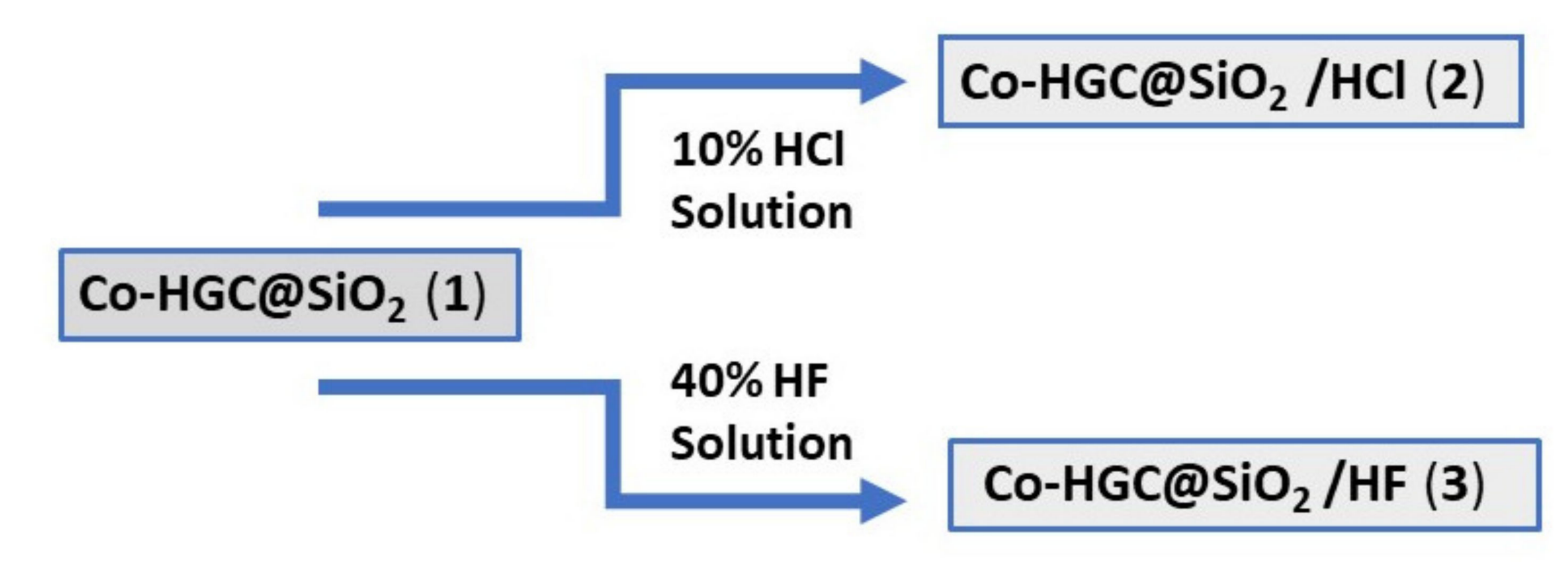
Schematic illustration of preparation of Co-HGC@SiO_2_/HCl (2) and Co-HGC@SiO_2_/HF (3) from Co-HGC@SiO_2_ (1).

Preparation of Co-HGC@SiO_2_/HCl (2): 20 mL of 10% HCl solution was added carefully to 0.5 g of (1) in a 50 mL reaction flask. Vigorous hydrogen gas evolution resulted. The reaction mixture was stirred for 30 min till gas evolution stopped. The excess acid solution was removed by filtration and the black powder was washed with water (3 ×X 10 mL). Then, the reaction mixture was collected and dried in air over to give 3.5 mg of (2), Scheme 2.

Preparation of Co-HGC@SiO_2_/HF (3): 1 mL of HF acid 40% was added dropwise to 5 mg of (1) in a 10 mL vial. Vigorous H_2_ and SiF_4_ gases evolution resulted. Caution should be taken as the reaction is violent. The mixture was agitated for 30 min till gas evolution stopped. Then, the excess acid solution was withdrawn with eye dropper. The obtained black powder was washed with water several time, then collected and dried in air oven to give 2.1 mg of (3), Scheme 2. 

Preparation of Co-HGC@SiO_2_/CV (4): 0.1g of (1) in 100 mL solution of crystal violet CV (300 mg/L) was agitated in an incubator shaker SI-300, GMI Inc, at room temperature for 24 h to ensure maximum adsorption of the dye. The black powder was filtered, washed with distilled water several time and dried in an air oven to give 0.11 g of (4). (Nanocarbon (4) was prepared in order to prove successful adsorption of CV dye onto (1)).

The methodology of preparation of different types of nanomaterials (1), (2) and (3) is presented in Scheme 1 and Scheme 2. Thus, (2) and (3) were prepared by chemical modification by treating (1) with acid HCl and HF solutions, while (4) was prepared by adsorption of crystal violet onto (1).

### 2.2. Instrumental Analysis

#### 2.2.1. BET Analysis

The nitrogen adsorption isotherm was measured with a Micrometrics 3Flex gas sorption analyzer (Micrometrics, Norcross, GA, USA) 30 mg of the sample was degassed at 85 °C for 36 h and backfilled with nitrogen gas. This temperature is good enough to remove the water molecules present in the pores. Adsorption isotherms were generated using incremental exposure to ultrahigh purity nitrogen up to 1 atm in a liquid nitrogen bath. Surface area values were calculated using Brunauer-Emmett-Teller (BET) equation included in the Micromeritics ASAP 2020 V4.00 software (Micromeritics, Norcross, GA, USA) suite.

#### 2.2.2. Powder X-ray Diffraction

Powder X-ray diffraction (PXRD) measurements were carried out using PAN analytical X’Pert PRO MP X-ray diffractometer(PANalytical, Almelo, The Netherlands) consisting of a focusing elliptical mirror and a fast-high resolution detector (PIXCEL) with the radiation wavelength of 0.15418 nm. Powder X-ray diffraction (PXRD) measurements were conducted with a Cu Kα (λ = 1.5405 Å) radiation source operating at 40 kV and 40 mA, and a divergence slit of 1/16° over the 2θ range of 5–90° with a step size of 0.01°.

#### 2.2.3. Raman Spectroscopy

Raman spectra were recorded with a HORIBA Jobin Yvon LabRAM HR Evolution Raman microscope (HORIBA, Palaiseau, France); Equipped with Syncerity scientific CCD Deep Cooled camera (HORIBA, Palaiseau, France) and an automated XY motorized stage. The line of 633 nm laser was used as the source of excitation. Before the measurements were performed, the micro-Raman spectrometer was well calibrated using 521 cm^−1^ Raman line of single crystalline Silicon (Si) wafer.

#### 2.2.4. Scanning Electron Microscopy

Morphologies of the synthesized samples are characterized by field emission scanning electron microscope (FE-SEM, JEOL JSM-76700F) (JEOL, Tokio, Japan) equipped with an EDX system (elemental mapping). All the samples are analyzed with acceleration voltage of 15 kV, working distance of 8 mm and sample vacuum of 1 × 10^−5^ Pa. Scanning electron microscopy imaging studies and energy dispersive X-ray analysis (SEM-EDX) were also performed on a FEI Quanta 450FEG high-vacuum electron microscope (FEI, Hillsboro, OR, USA) equipped with an EDX system (elemental mapping) from EDAX.

#### 2.2.5. High Resolution Transmission Electron Microscopy (HRTEM)

HR-TEM images of samples were obtained using a Talos F200X Scanning/Transmission Electron Microscope (STEM) (Thermo Fischer, Waltham, MA, USA) with a lattice-fringe resolution of 0.14 nm at an accelerating voltage of 200 kV equipped with CETA 16M camera (Thermo Fischer, Waltham, MA, USA).

#### 2.2.6. X-ray Photoelectron Spectroscopy (XPS)

The measurements were carried out on SPECS GmbH high vacuum multi-technique surface analysis system (SPECS, Berlin, Germany) supplied with an Al-Kα 1486.7 eV X-ray source. Calibration of the spectra was achieved by setting C1s line at 284.8 eV.

#### 2.2.7. Elemental Analysis

Elemental composition especially carbon, nitrogen, hydrogen and sulfur composition of the nanocarbon sample before and after acid treatment were examined by Vario microcube elemental analyzer (LabX, Ronkonkoma, NY, USA) under CHNS mode.

#### 2.2.8. Adsorption Methodology

##### Effect of Silica Support

In order to evaluate the effect of silica support in the nanocomposites on the adsorption efficiency, (5) was prepared with no silica and its capacity was compared to the capacity of the product obtained with silica (1). Thus, cobalt hierarchical graphitic carbon nanoparticles Co-HGC (5) was prepared similarly to Co-HGC@SiO_2_ (1), by pyrolysis at 850 °C of cobalt phenanthroline sulphate complex with pyrene (1:1) but with no addition of silica. 

##### Effect of Concentration, Isotherms

The same experimental condition was used for the effect of concentration and isotherm studies. Adsorbent mass (m = 0.01 g) was added to dye solutions with initial concentration C_i_ = 25, 50, 100, 150, 200, 250, 300, 350 mg/L (volume = 10 mL). The mixture was shaken at room temperature for 24 h, then the remaining final concentration of the dye C_f_ (mg/L) at equilibrium was calculated from the measured absorbance of solution. The % removal of dye and the capacity q (mg/g) (the amount of solute adsorbed per unit mass of adsorbent) can then be calculated.

##### Effect of Time

The following experimental condition was used to study the effect of time. The mass of adsorbent m was 0.01 g. The initial concentration of dye *C_i_* was 100 mg/L and the volume of solution V = 0.05 L. The remaining concentrations of dye *C*_f_ (mg/L) were measured at different time t (min) up to 24 h. The adsorption capacity q (mg/g) was then calculated at each time. 

##### Effect of pH

In a typical experiment, 0.01 g of adsorbent in 10 mL of dye solution MO (100 mg/L) or CV (50 mg/L) was shaken for 24 h at different pH. The remaining concentration of dye C_f_ (mg/L) was then measured. The equilibrium adsorption capacities qe (mg/g), and % removal of CV and MO by nanocomposites (1), (2) and (5) were measured at different pH (3, 5, 7, 11 and 13). 

## 3. Results and Discussion

[Co(phen)(H_2_O)_4_]SO_4_·2H_2_O complex was pyrolyzed in the presence of pyrene and silica in the ratio (1:1:1) under nitrogen atmosphere in an inert gas furnace up to 850 °C. The product was silica-supported hierarchical graphitic carbon sheltering cobalt nanoparticles Co-HGC@SiO_2_ (1), Scheme 1. The expected complete loss of coordinated and crystalline water from [Co(phen)(H_2_O)_4_]SO_4_·2H_2_O complex occurred at about 200 °C. The loss of phenanthroline ligand is expected to occur in the temperature range 300 °C–500 °C, [33]. Thus, heating the complex at an intermediate stage 300 °C for 2h ensures loss of water and some of phenanthroline ligands and promote the synthesis of cobalt nanoparticles in situ. The later can act as catalyst for graphitization of carbon atoms which originates from decomposed phenanthroline and pyrene in the gaseous state at above 500 °C. Complete graphitization by heating at the optimum temperature 850 °C for 15 h was concluded by analyzing SEM of pyrolyzed products at different temperatures and times.

### 3.1. Characterization of Nanocomposites

#### 3.1.1. SEM and EDX of Nanocomposites

**Figure 1:**
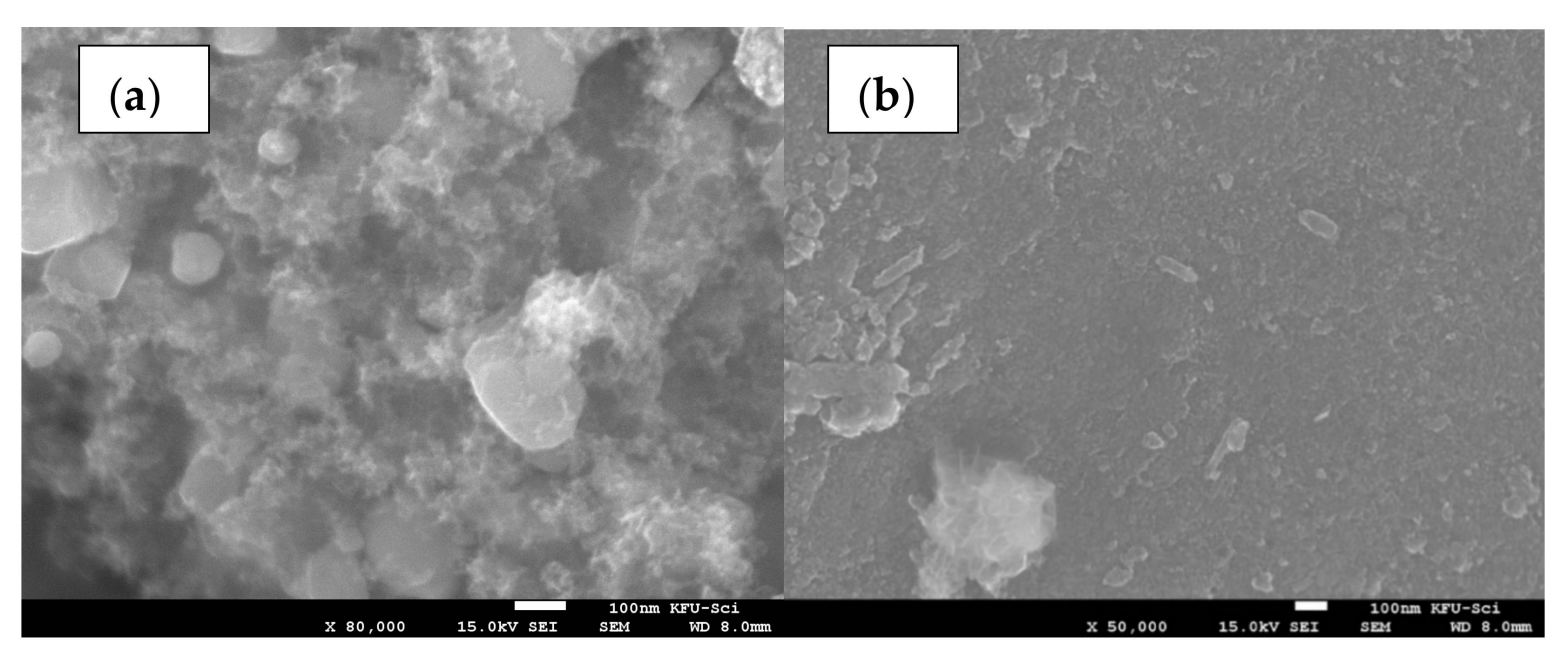

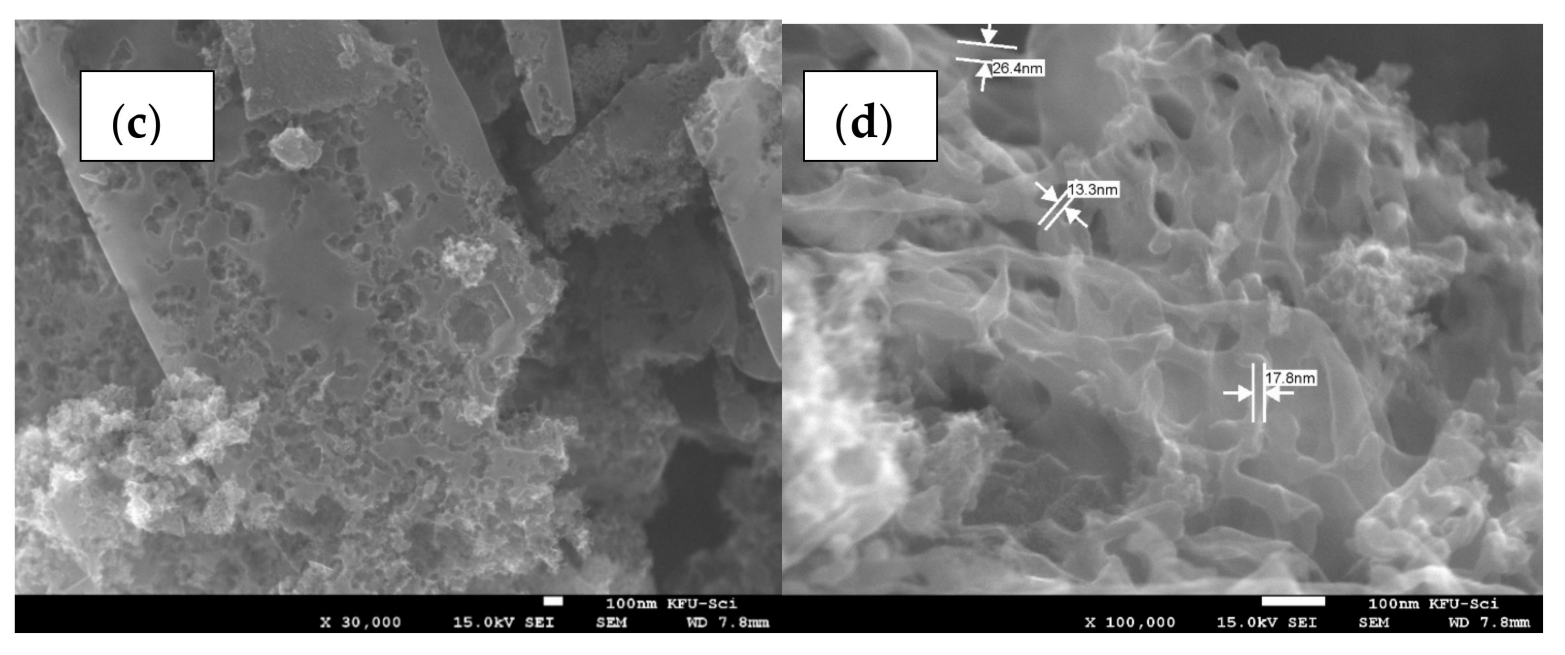
SEM analysis of nancomposites; (**a**) SEM of (1), (**b**) SEM of (2), and (**c**) SEM of (3) and (**d**) SEM of (3).

**Figure 2:**
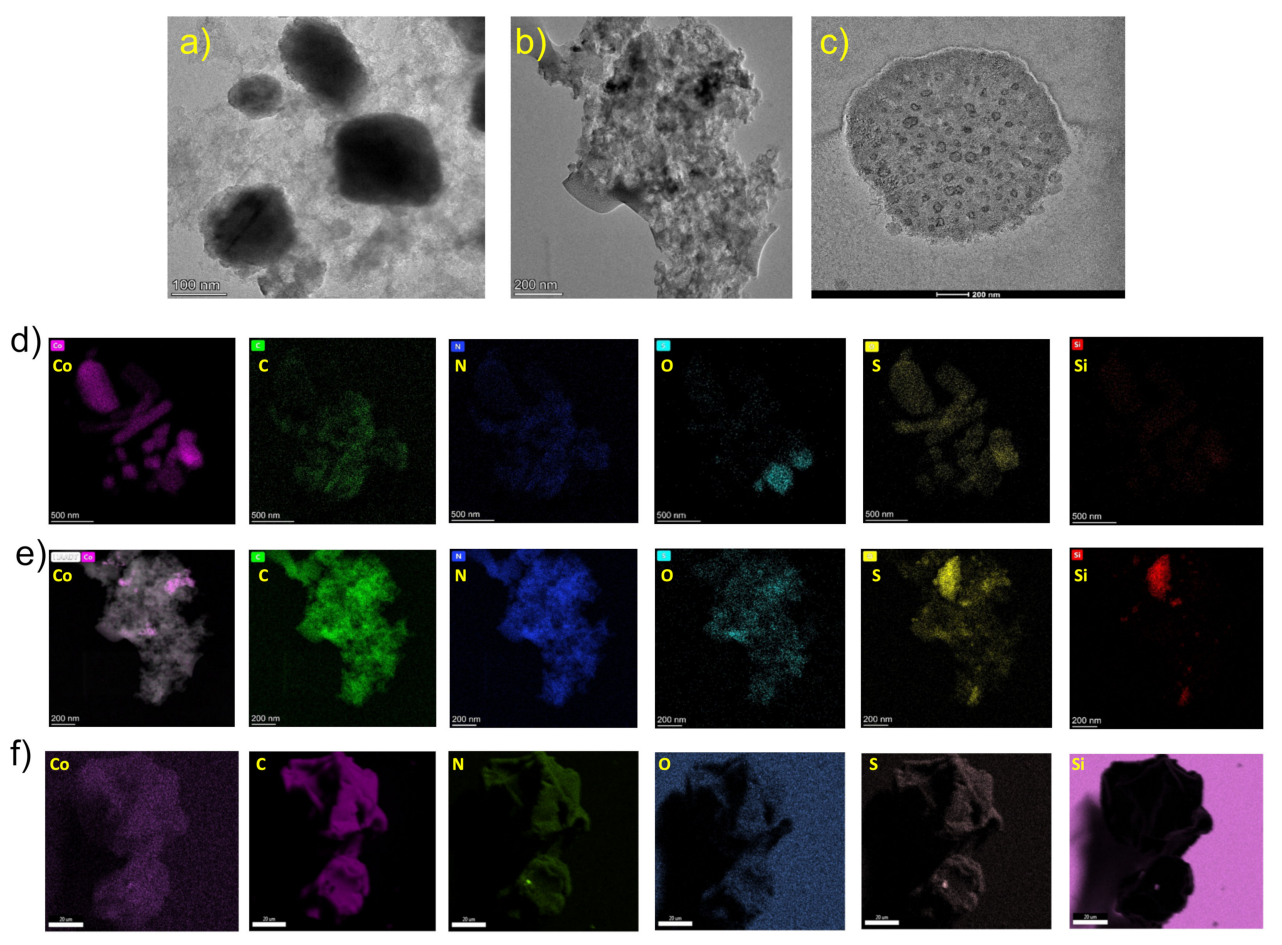
TEM images, and EDS-HAADF mapping of nanocomposites (1), (2) and (3); TEM analysis of (1) (**a**), (2) (**b**), and (3) (**c**); EDS-HAADF mapping of (1) (**d**); EDS-HAADF mapping of (2) (**e**); EDS-HAADF mapping of (3) (**f**).

The size and morphology of silica-supported hierarchical graphitic carbon sheltering cobalt nanoparticles CoHGC@SiO_2_ (1, 2 and 3) was probed using scanning electron microscopy SEM as shown in Figure 1 and Figure 2. It is clear that the silica support is covered with spongy carbonaceous matrix enclosing white cobalt nanoparticles. SEM with higher magnification clearly shows that the surface of nanocomposite (1) consists of hierarchical 3-D carbon sheltering irregular cobalt nanospheres of diameter 50 to 200 nm, Figure 1a. The nanocomposite (2) still shows graphene nanosheet with rough surface due to loss of cobalt/cobalt oxide nanoparticles after the addition of 10% HCl acid treatment of (1) Figure 1b. The SEM image of nanocomposite (3) showed spongy platelets with nanopores caused by dissolution of silica support as a result of HF treatment of (1), Figure 1c. It is noteworthy to mention that cobalt nanoparticles were also lost from the structure by HF treatment. SEM high resolution image of (3) shows irregular tubules with diameter 10×30 nm and length of order of hundreds of nanometers, Figure 1d. Cobalt nanocarbon CoHGC (5) prepared similarly to (1) but without the addition of silica, showed similar morphology to (1) as indicated by SEM of (5), Supplementary Figure S1.

From the above observations, thermal treatment causes decomposition of cobalt complex at low temperature about 300 °C, where cobalt ions combine together to form cobalt nanoparticles which act as graphitization catalysts. The phenanthroline ligand and the extra carbon source anthracene both were carbonized at 400–410 °C. Higher temperature up to 850 °C was necessary to condense 3-D Hierarchical carbon on the silica support with the aid of cobalt nanoparticles. 

Table 1 presents the EDX composition of samples (1), (2) and (3). The O/Si ratio of atomic% composition of 3.14 in (1) can thus be calculated, (see Supplementary Materials, Figure S2). The expected ratio calculated from SiO_2_ is only 1.14. Therefore, the excess oxygen is present as functional group within the carbon matrix and as sulfur oxide and cobalt oxide. While the % atomic ratio for O/Si in (2) is 2.67. Thus, excess oxygen is only found in carbon matrix as functional groups, since the atomic % of S and Co are negligeable in (2). The atomic% S (3.54) and % Co (8.19) in (1) decreases to 0.47 and 0.56 in (2) due to treatment with acid HCl. While in (3) the atomic% of carbon increases to 85.65% at the expense of Co and Si which decrease to 4.29% and 0.047% due to treatment of (1) with HF acid. 

#### 3.1.2. TEM and EDS of Nanocomposites

The microstructure of all the nanocomposites was further characterized by high-resolution transmission electron microscope TEM. Nanocomposite (1) consists of silica particles coated with graphitic carbon decorated with cobalt/cobalt oxide nanoparticles (Figure 2a). The TEM image shows cobalt/cobalt oxide irregular nano spheres of several 100 nm in length and diameter. The nanoparticles are surrounded by hierarchical carbon microlayers. EDS-HAADF images also show cobalt irregular nano rods and spheres. Both cobalt and oxygen elements are fully enriched in nanocomposite (1). Carbon, nitrogen, silicon and sulfur are spread over the same locations. The elemental mapping EDS-HAADF of (1), gave C 32.11% with a high loading of cobalt (31.66%) in the nanocomposite (Figure 2a).

TEM image of (2) shows some white pores of size 10–50 nm for which cobalt nanoparticles were extruded due to HCl treatment of (1) (Figure 2b). Few black cobalt (0) nanoparticles were left. They resisted acid attack most likely because they are encapsulated by graphite matrix. EDS-HAADF analysis of (2) reveals small pink spots for cobalt, while carbon, nitrogen and sulfur are spread over the whole area of nanomaterials. This suggests that N and S form bonds with carbon in the matrix. The high oxygen populated zone is associated with the silicon zone. Thus, they belong to silica support (Figure 2b). TEM-EDAX measurement of (2) show low atomic % Co (0.19%) similar to the EDX measurement, indicating loss of cobalt. Meanwhile, % contents of SiO_2_ support and carbon matrix are not affected by HCl treatment of (1) (Figure 2b).

Finally, TEM analysis of (3) shows a leftover of a silica particle about 300 nm in diameter coated with a carbon matrix (Figure 2c). The inner silica was lost due to HF treatment of (1) leaving behind empty pores with a dark border, and no cobalt nanoparticles were observed. Few carbon lattice fringes were observed only at the border of the nanostructures. EDS-HAADF mapping results are in agreement with TEM-EDAX elemental mapping results.

#### 3.1.3. Raman of Nanocomposites

**Figure 3:**
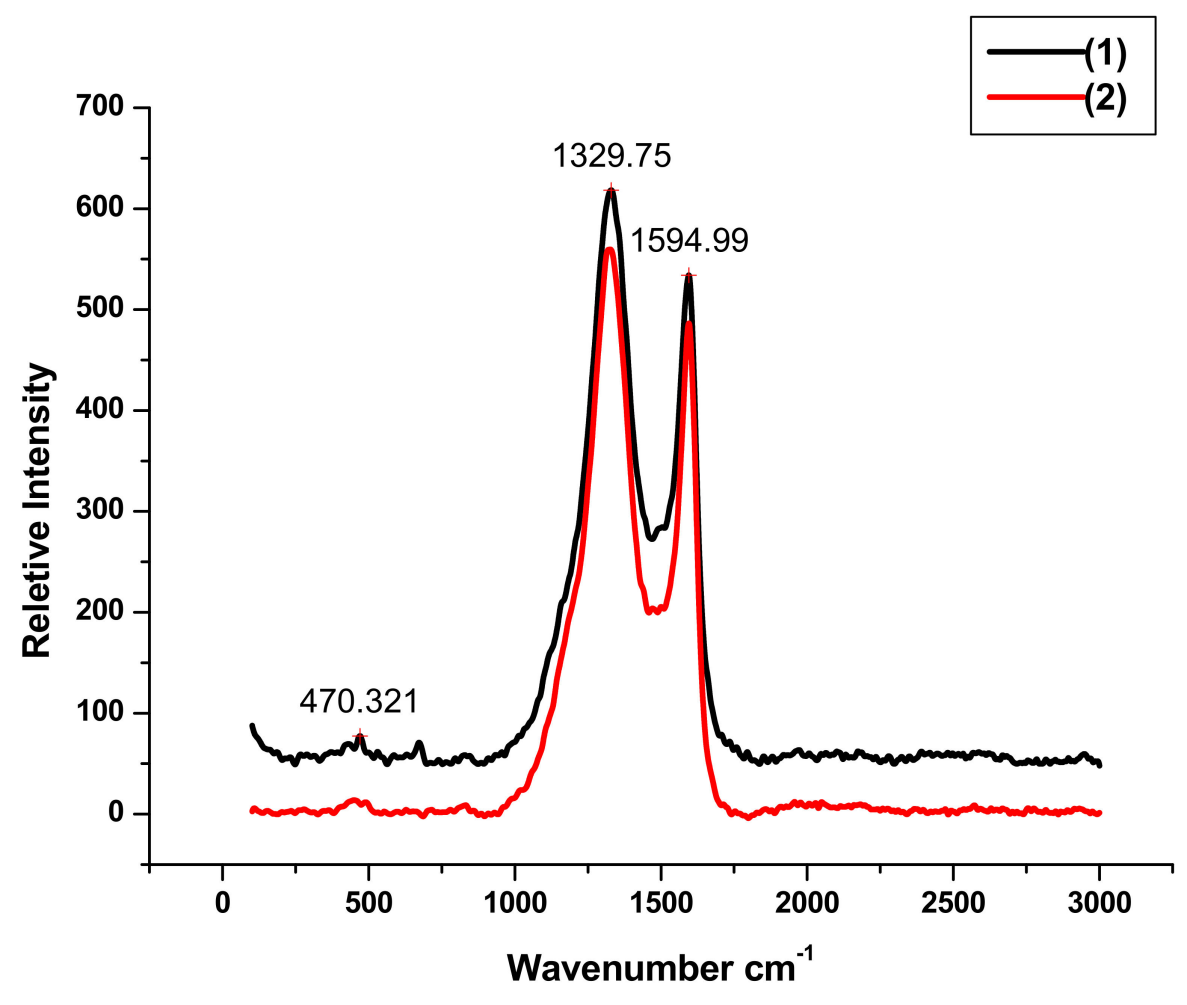
Raman spectra of (1) and (2).

Additional information about the structural properties of the nanocomposites (1) and (2) were obtained from Raman spectroscopy, Figure 3. The sharp peaks located at 1329.75 and 1594.99 cm^−1^ can be attributed to D-bands and graphitic G-bands of carbon respectively. The ratio of intensities I_D_/I_G_ is a measure of defect present in graphene structure. The I_D_/I_G_ ratios 1.17 for (1) and 1.15 for (2) are almost the same. This indicates that the microstructure was not affected by acid treatment. The peaks at 470.32 and 675.87 cm^−1^ in Raman spectra of (1) and (2) are characteristic of cobalt monoxide CoO, [38]. The intensities of these peaks were drastically decreased in the case of (2) due to dissolution of cobalt oxide as a result of HCl acid treatment. 

#### 3.1.4. PXRD of Nanocomposites

**Figure 4:**
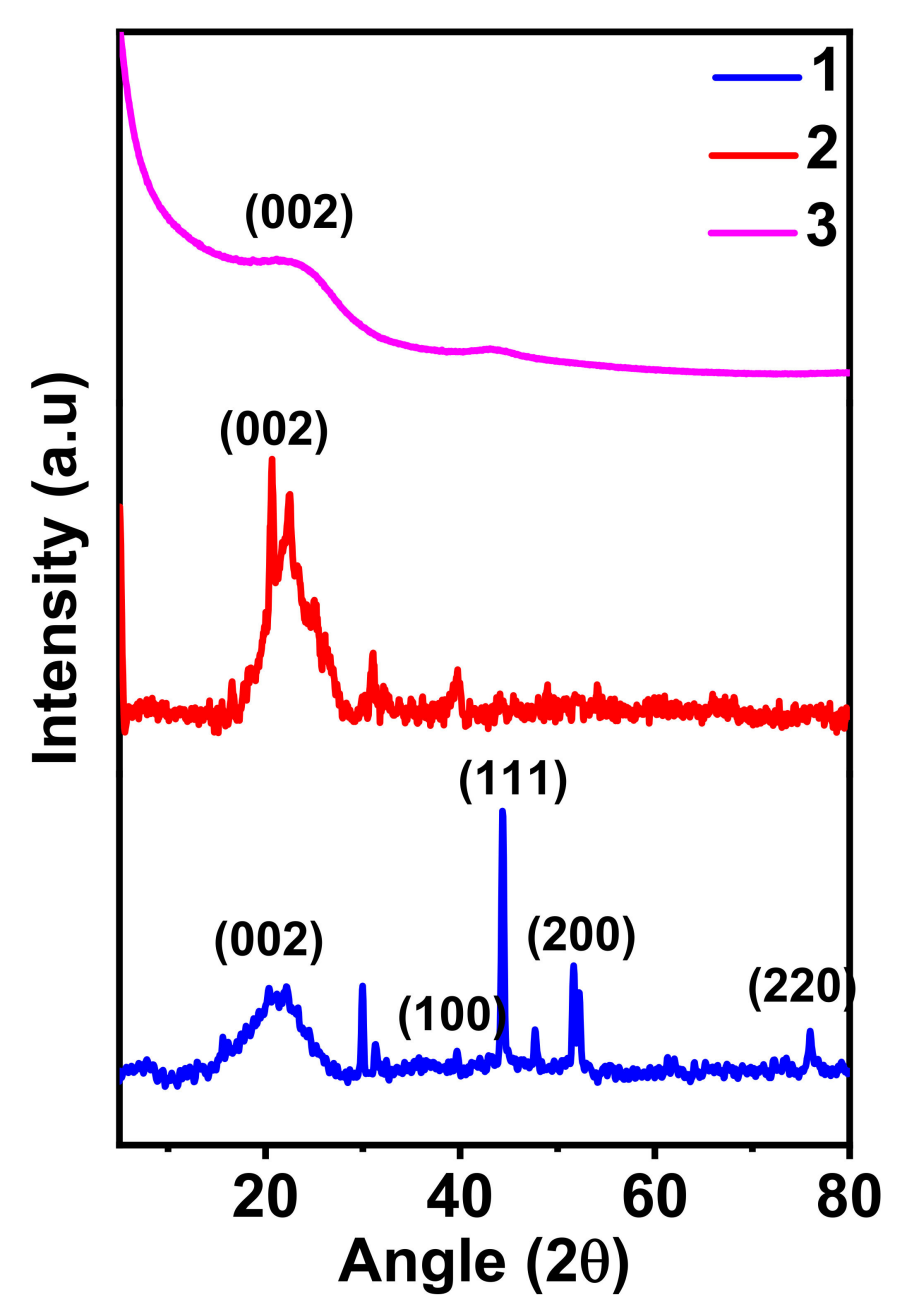
Powder X-Ray diffraction patterns of nanocomposites (1), (2) and (3).

The crystalline nature of the cobalt silica nanocarbon samples (1), (2) and (3) were examined using powder X-ray diffraction studies at room temperature. The nanocomposite (1) exhibits two board distinct peaks at 20.50° and 22.30° corresponding to (002) planes of graphitic carbon (Figure 4). Other peaks are observed in PXRD pattern of sample (1) at: 30.1, 31.5, 44.4, 47.9, 52.1, 52.6 and 76.1°. The experimental 2θ values at 44.4, 52.1, and 76.1 correspond to the theoretical 2θ values for metallic cobalt nanocarbon particles (111), (200), and (220) planes respectively and match with (PDF # 15-0806), [39,40]. Two broad peaks were observed at 22.33 (strong) and 44.17 (medium). They can be assigned to (002) and (100) of graphite carbon, respectively [41,42]. After treatment of sample (1) by HCl, the cobalt nanoparticle peaks at 44.4, 47.9, 52.1, 52.6 and 76.1° disappeared in nanocomposite (2). PXRD pattern and graphite carbon peak at around 20.8° was unaffected. Alternatively, the addition of HF to sample (1) caused a loss in the crystalline nature of (3) and gave a broad diffraction pattern characteristic of graphite carbon. Moreover, nanocomposite (3) lost the silica characteristic diffraction peak at 20.69 (JCPDS PDF No. 46-1045) [43]. A broad diffraction peak at 2θ = 18–22° confirms the presence of amorphous graphitic carbon. 

#### 3.1.5. Zeta Potential of Nanocomposites

In order to gain insight into surface charge of nanomaterials, dynamic light scattering (DLS) measurements were performed at room temperature on (1), (2) and (3) using Malvern Zetasizer Nano Series. The ζ-potential (Zeta potential) values were presented in Supplementary Table S1.

Change in pH of the medium causes a variation of electric charge in the tern layer and, consequently, variation in E(mV) electric potential. At neutral pH, the zeta potential of nanocomposite (1) is −1.6 mV. At lower pH, the zeta potential becomes more positive, owing to the dissolution of cobalt nanoparticles and giving cations in the acid medium. On the other hand, in basic medium at higher pH 14, the zeta potential becomes more negative due to the dissolution of some silica and conversion of cobalt nanoparticles to negatively charged cobalt hydroxide. The same zeta potential trend is reflected in the case of nanocomposites (2) and (3) at both low and high pH. However, (2) and (3) contained fewer cobalt nanoparticles than (1) due to dissolution of cobalt by HCl acid. Furthermore, the silica content of (3) is very much less than that of (1) and (2), due to dissolution of silica by HF acid during the preparation of (3). Additional contribution to positive potential comes from the effect of protonation of nitrogen, sulfur and oxygen groups on the graphitic carbon matrix at low pH in all samples. Additional contribution to negative potential at higher pH can be due to the adsorption of hydroxyl ions onto the surface of nanocarbons.

#### 3.1.6. XPS of Nanocomposites

The XPS spectra for (1), (2) and (4) are presented in Supplementary Figure S3. The elements Co, O, N, C and Si were found on the surface of nanocomposites. Briefly, the XPS elemental composition indicated that Co is only 0.74% in (2) since HCl treatment dissolved and removed most cobalt species form (1). The adsorbed crystal violet CV contributes to increase the elemental composition % C (46.72) and % N (1.30) and decrease the elemental % Si (4.55) in the surface of (4) compared to other samples.

XPS convolution spectra of cobalt 2p for the samples were shown in Supplementary Figure S4 and the fitting indicates the presence of CoO species [44]. For (4), the Co2p3/2 and Co 2p1/2 spin orbit splitting is 16.0 eV, while the shakeup lines splitting is 16.6 eV. This is well matched with already reported values of 15.9 eV and 16.5 eV [45]. 

**Figure 5:**
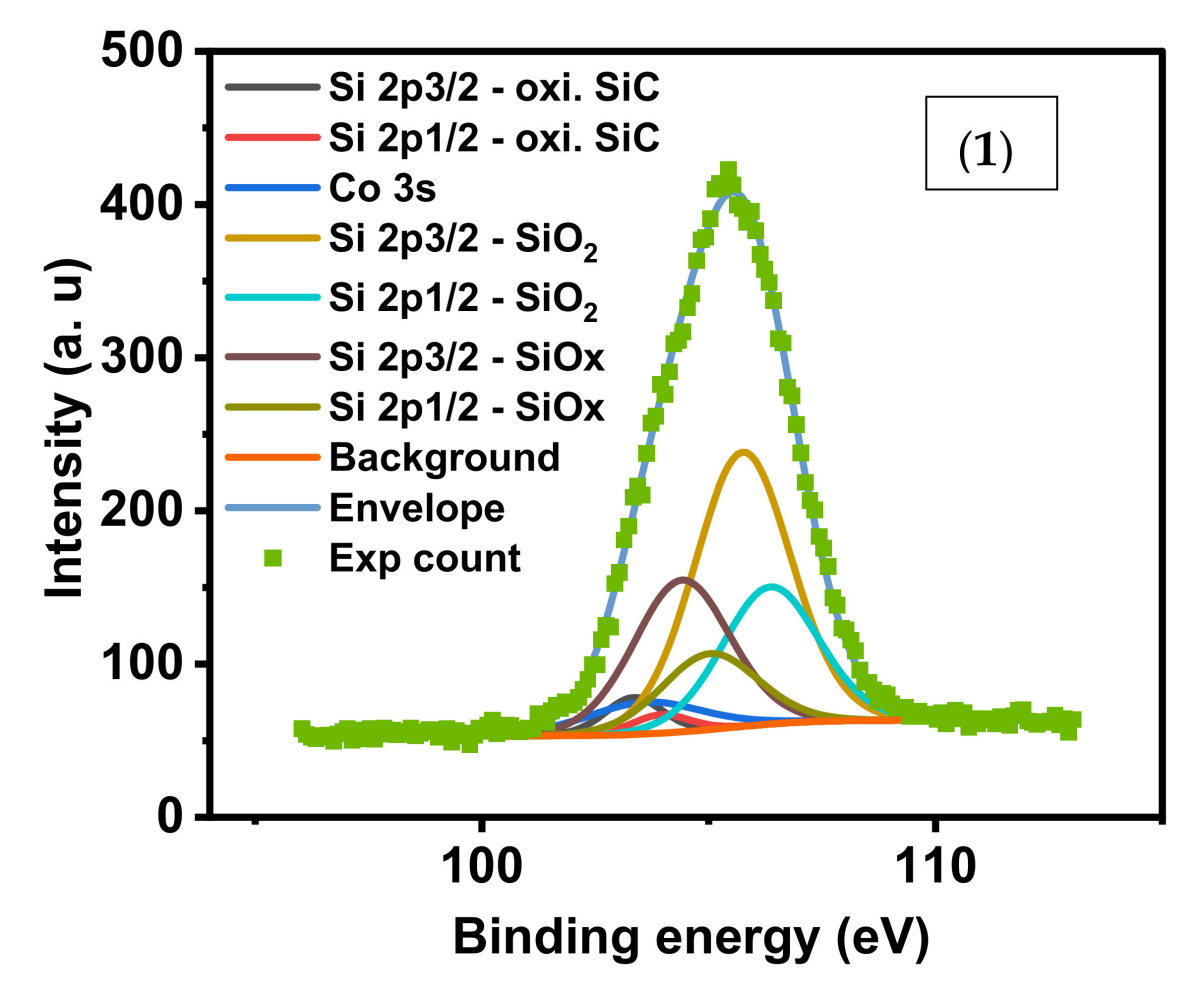

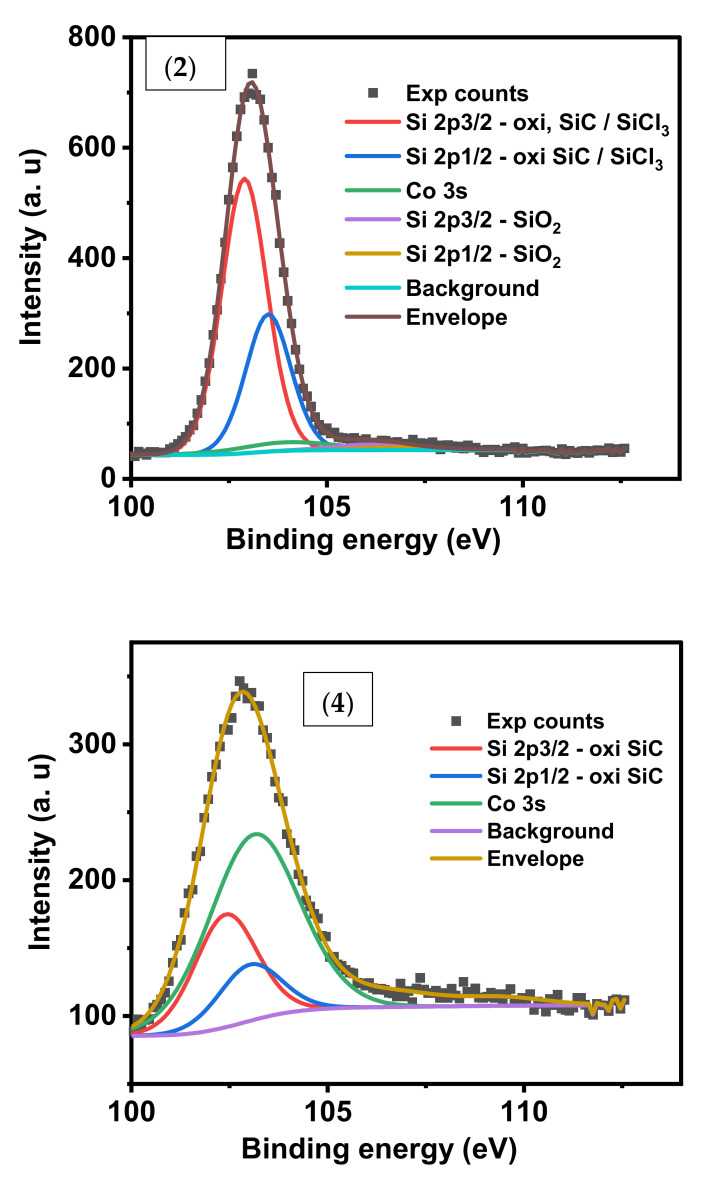
XPS convolution Si2p-Co3s spectrum for the three samples (1), (2) and (4).

In the Si2p/Co3s region, the SiO_2_ and SiO_x_ peak is highest for (1), Figure 5. Nanocomposite (2) showed highest amount of oxidized SiC and SiCl_3_ due to treatment of (1) with HCl during its preparation. XPS peaks of SiO_2_ is negligible in (2) and absent in (4) due to effective surface adsorption of crystal violet. The Co 3s in sample (4) was observed at 103.13 (40.68%), 106.63 (3.17%), and 109.43 (1.86%). The contribution of Co 3s to the same region in (1) and (2) was less than 3%.

The O1s XPS convolution spectrum (Supplementary Figure S5) revealed that the main peak refers to O=C species more than oxidized SiC and carbonates. This peak is highest for sample (2) with 82.47% contribution to total oxygen. 

**Figure 6:**
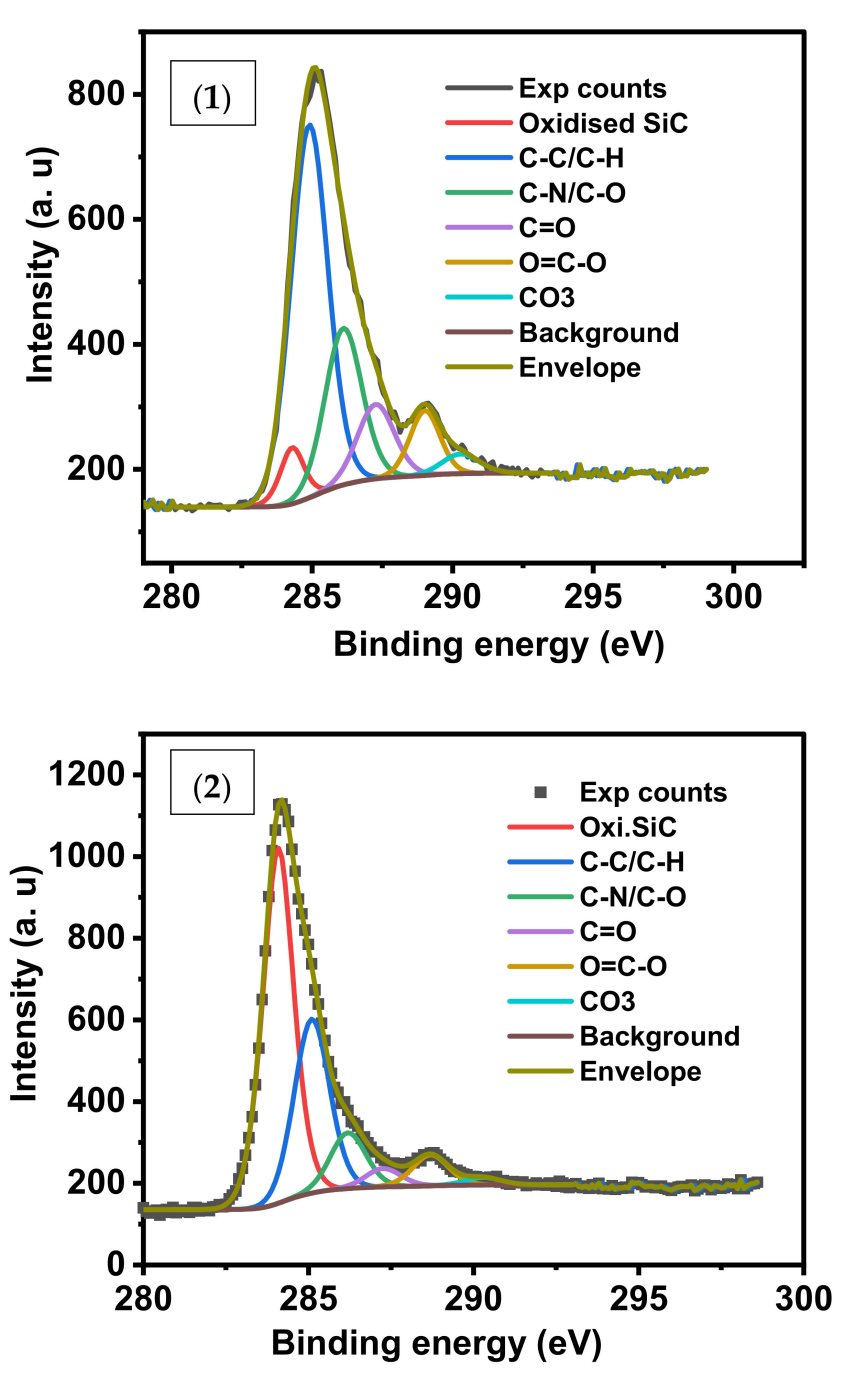

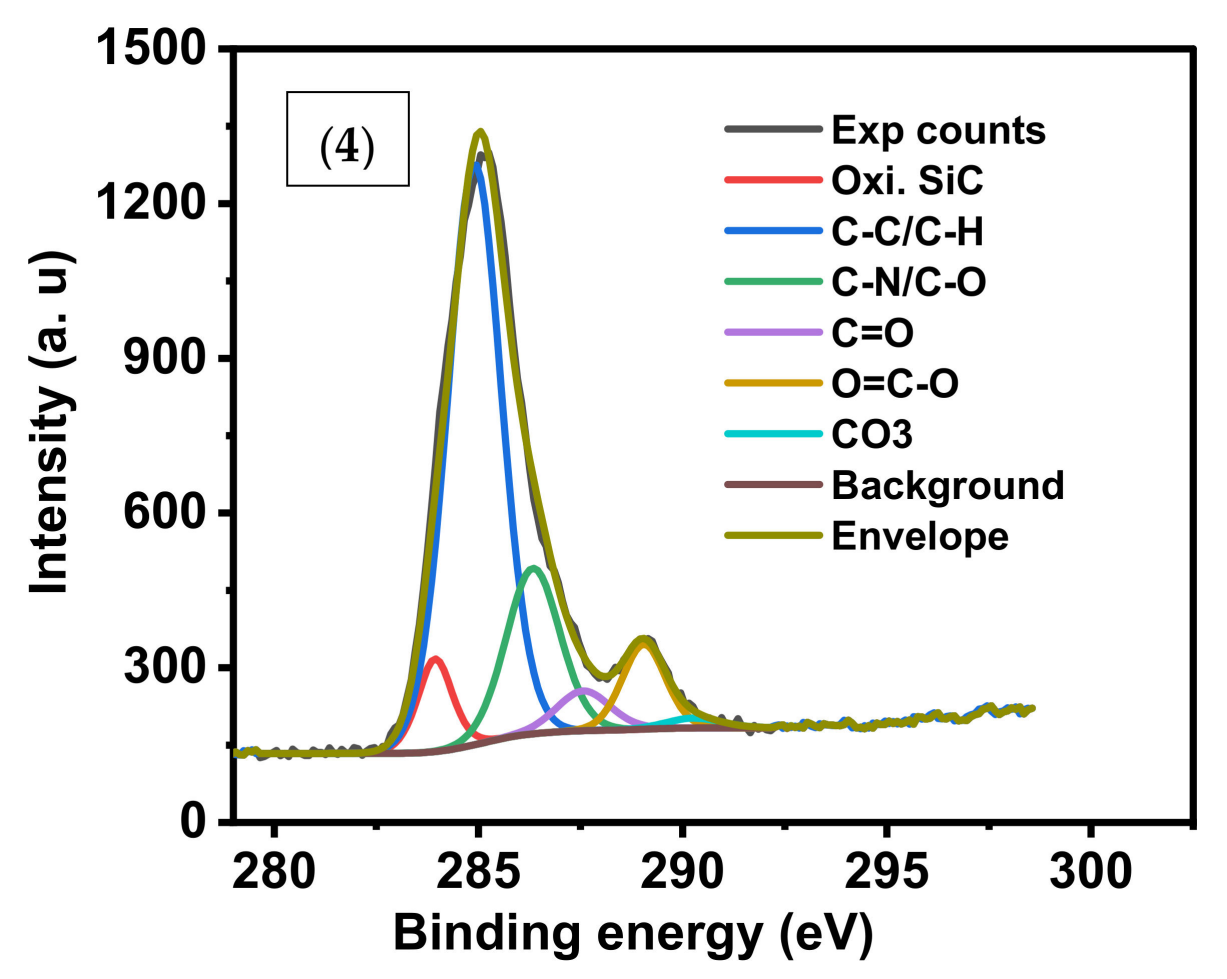
XPS C1s deconvoluted spectrum for samples (1), (2) and (4).

The XPS C1s convolution spectra for the three samples (1), (2) and (4) were shown in Figure 6. The oxidized SiC species in sample (2) represent 52.39%, the highest percentage contribution of carbon species among the samples. C-C/C-H carbon constitutes 62.7% in (4) which indicates efficient adsorption of CV by sample (1). 

**Figure 7:**
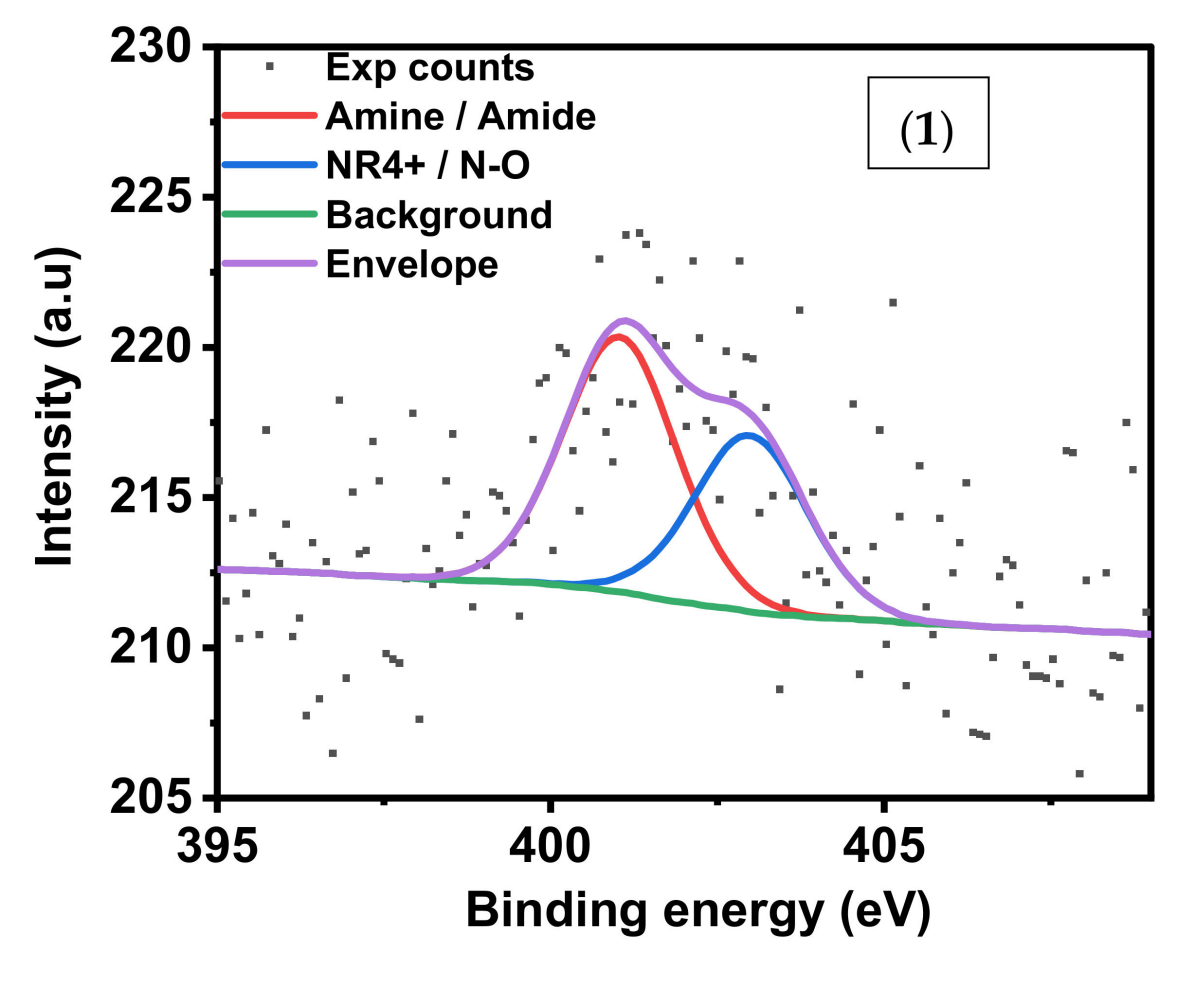

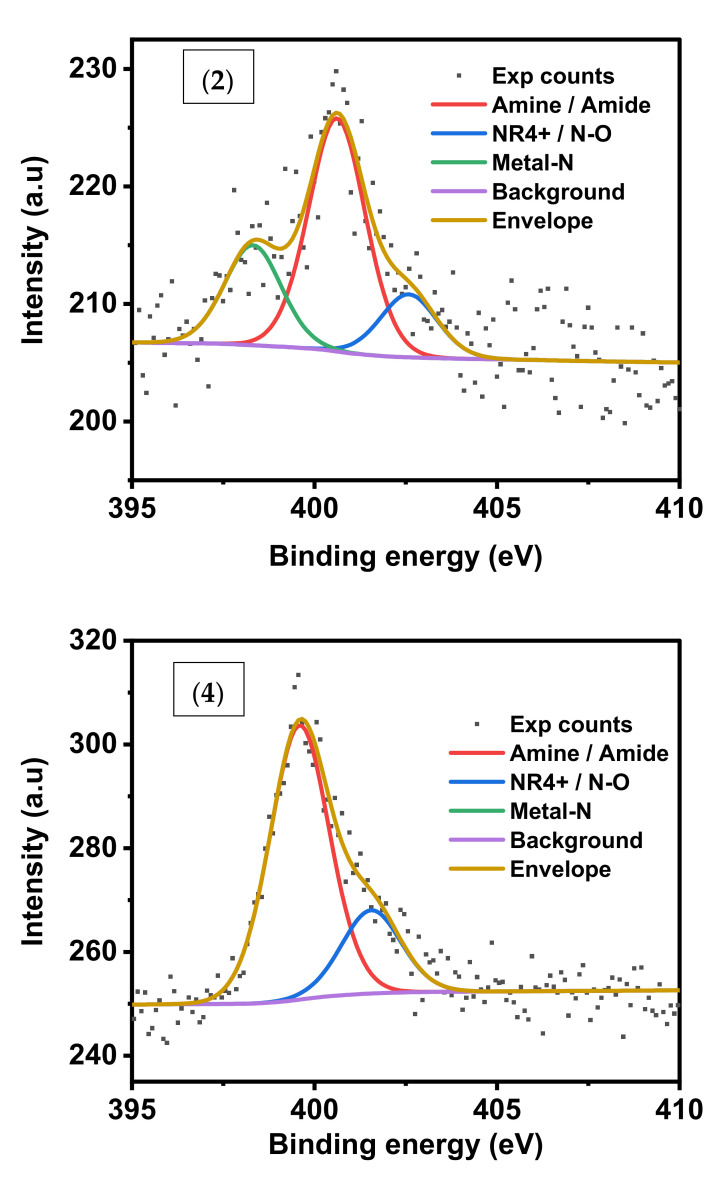
XPS convolution N1s spectrum for the three samples (1), (2) and (4).

The amine/amide contribution is 59.1% in the N1s spectrum of (1) and 58.87% for (2). It increased to 76.69% for (4) due to the presence of adsorbed CV dye which contains amine groups, Figure 7. Moreover, Co-N constitutes 25.23% in the N1s spectrum of (2) reflecting that cobalt nitride species is formed during thermal treatment of cobalt complex.

#### 3.1.7. Surface Area Analysis of Nanocomposites

**Figure 8:**
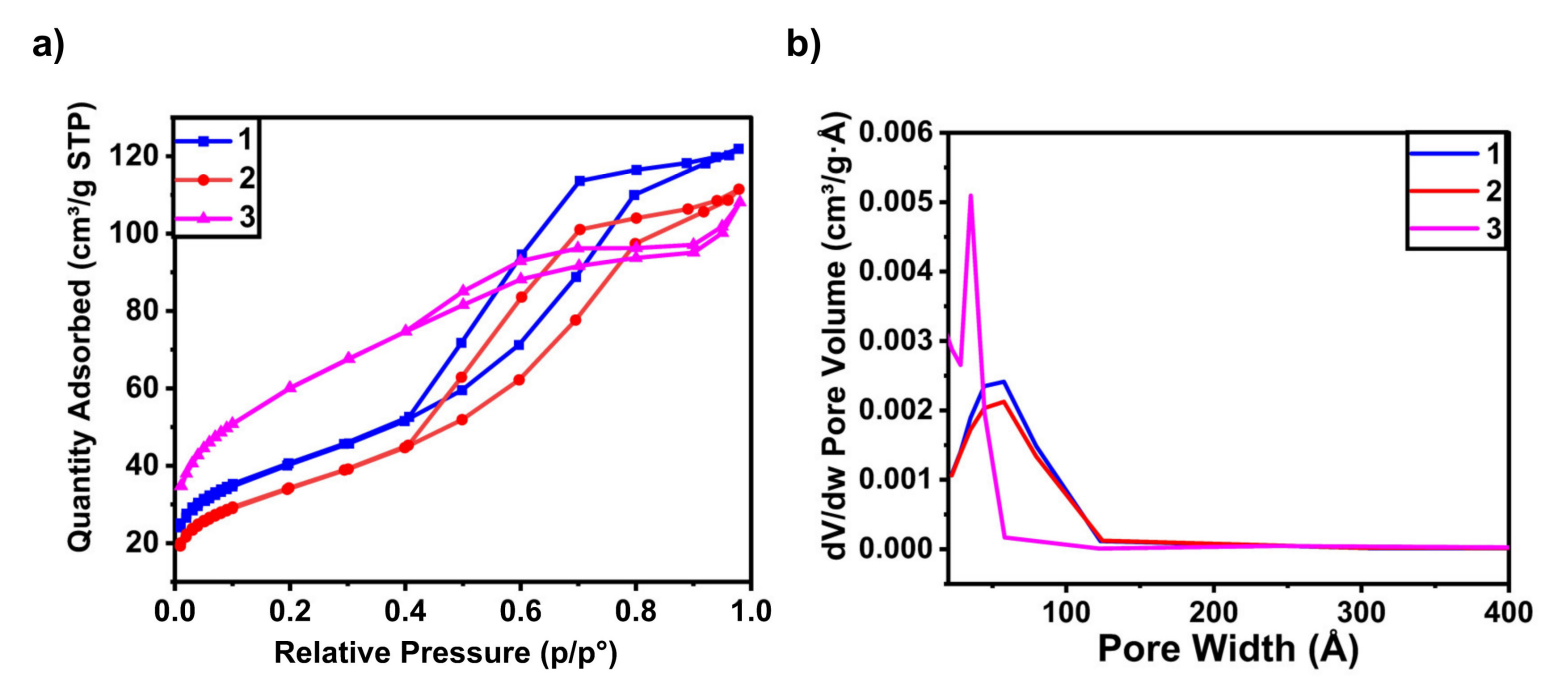
(**a**) N_2_ adsorption/desorption isotherms at 77K of samples (1), (2) and (3); (**b**) their corresponding BJH mesopore size distribution curves of the samples (1), (2) and (3).

Brunauer–Emmett–Teller (BET) surface areas and Barrett–Joyner–Halenda (BJH) pore sizes and volumes of nanocomposites 1, 2 and 3 were determined. The surface areas of (1), (2) and (3) were measured using N_2_ adsorption/desorption at 77K after degassing the samples at 85 °C for 36 h. The corresponding sorption isotherms are shown in Figure 8a. The isotherms were fitted to the BET model. The obtained BET surface areas were 144.8, 123.5 and 217.5 m^2^g^−1^ for (1), (2) and (3) respectively, with a typical type-IV and H2 (b)-type hysteresis loop according to IUPAC classification. H2 (b) type hysteresis arises a narrow distribution of pore bodies with a wide neck size distribution, leading to a steep desorption step in the isotherm via cavitation. Furthermore, the hysteresis loops in the *P*/*P*_0_ range from 0.40 to 0.98 for both adsorption and desorption isotherms, further confirming the formation of mesopore and macropores in the samples. The pore size distributions of the samples were calculated using the BJH method. The curves are shown in Figure 8b. The pore size distribution plot showed narrow mesopore and macropores size distribution, with an average pore size of 51.3, 52.5 and 40.5 Å for samples (1), (2) and (3), respectively. The pore volume ranges of the three samples are 0.176–0.195, 0.165–0.178 and 0.118–0.142 cm^3^g^−1^, respectively. All BET/BJH sorption measurement results suggest that the addition of HCl to sample (1) helped to remove 8% Co of metal present in (1). This caused a slight deformation of the mesopore structure and a 14% decrease in the BET surface area of (2). On the other hand, addition of HF to nanocomposite (1) leads to the removal of silica and some amounts of cobalt species. This leads to a decrease in pore size and an increase in the surface area of sample (3).

### 3.2. Adsorption Conditions

#### 3.2.1. Effect of Time

The capacity q (mg/g) at time t can be calculated from q = (C_i_ − C_f_) × V/m. While the % removal of dye can then be calculated according to % dye removal = (C_i_ − C_f_) × 100/C_i_.

For the system (2)-MO, 71% of the dye is removed after only 30 min, while for (1)-MO it is 36 % at 30 min then increased to 58% after 240 min. While for (1)-CV the % removal is 48% after 240 min, and for (2)-CV % removal is 41% at 240 min. In all cases the uptake of dye becomes slow after 240 min. The maximum % removal is obtained after 24 h. Thus, for isotherm studies the adsorption experiment was left for 24 h to ensure complete adsorption at equilibrium.

#### 3.2.2. Effect of Concentration

Adsorption isotherms explain how adsorbates interact with an adsorbent. Figure 10a represents the effect of initial concentration on adsorption equilibrium of dyes. It was observed that the % removal is highest for small initial concentration of dye, then it decreases with increasing initial concentrations. The % removal of CV dye by adsorbents (1) and (2) decreases from 91% and 92% for the solution with concentration 25 mg/L, to 48% and 28% for solution (350 mg/L) respectively. While the % removal of MO dye by adsorbents (1) and (2) decreases from 67% and 73% to 40% and 47% respectively, for solutions with the same range of concentrations. For adsorbent (5) in going from 25 mg/L to 250 mg/L of CV dye solution, the % removal decreased from 56% to 38%; while in the case of MO dye the % removal decreased from 84% to only 80% indicating higher efficiency of uptake for MO by (5).

#### 3.2.3. Effect of pH

**Figure 9:**
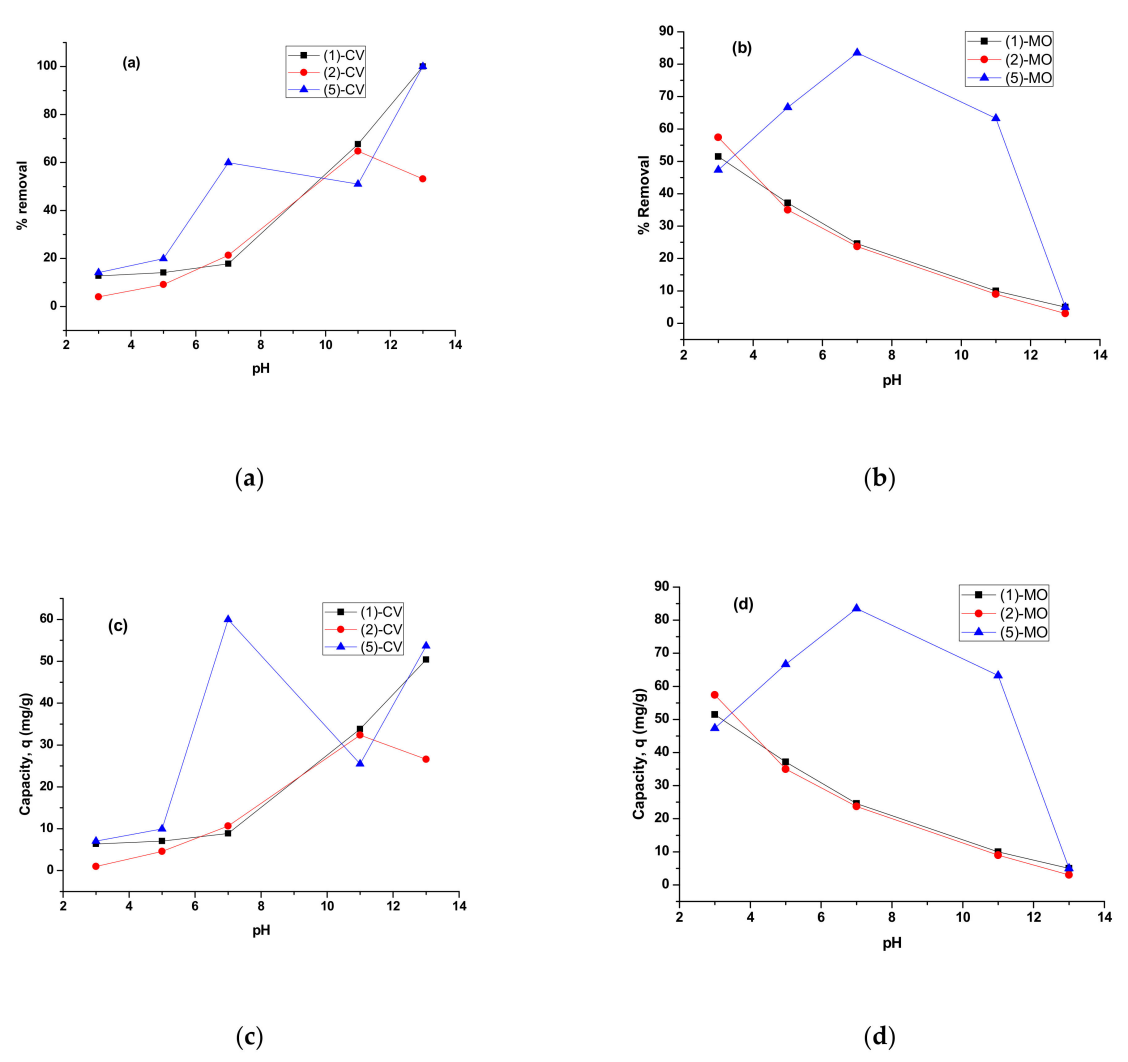
% removal of CV and MO dyes (**a**) and (**b**) and capacity q (mg/g) (**c**) and (**d**) by nanocomposites (1), (2) and (5) at different medium pH. (0.01 g adsorbent in 10 mL of dye solution MO (100 mg/L) or CV (50 mg/L), after 24 h).

The data is represented in Figure 9a,b. It is clear that the % removal of CV by all adsorbents increased from low to high pH, but with interference from decomposition of CV in strongly basic medium. The % removal of MO decreases from low to high pH medium, except with the adsorbent (5) where the maximum occurs at pH = 7. The capacity q (mg/g) follows the same trends as % removal, Figure 9c,d.

All three nanocomposites (1), (2) and (5) showed higher uptake of CV at highly basic pH due to electrostatic interaction between cationic/neutral dye and negatively charged nanocomposites. The anionic nature of nanocomposites at higher pH, basic medium was confirmed by zeta potential measurements. While minimum uptake of MO is reached at high pH due to repulsion between MO anions and negatively charged nanocomposites. On the other hand, all three nanocomposites (1), (2) and (5) showed considered high uptake of MO up to 60% at lower pH (acidic medium) due to interaction between neutral MO and cationic nanocomposites. The zeta potential measurement of the all nanocomposites confirmed the cationic nature of nanocomposites. While minimum uptake of CV occurs at low pH due electrostatic repulsion between similar positively charged nanocomposites and CV.

### 3.3. Adsorption Isotherm

Two famous Langmuir and Freundlich models were employed to describe the experimental results of adsorption isotherms.

Langmuir linear equation:

(1) $$\frac{Ce}{qe}=\frac{Ce}{q_{\max}}+\frac{1}{K_{L}q_{\max}}$$

Langmuir nonlinear equation:

(2) $$q_{e}=\frac{q_{\max}K_{L}C_{e}}{1+K_{L}C_{e}}$$

Freundlich linear equation:

(3) $$\ln q_{e}=\ln K_{F}+\frac{1}{n}\ln C_{e}$$

Freundlich nonlinear equation:

(4) $$qe=K_{f}{\left(Ce\right)}^{\frac{1}{n}}$$

The adsorbent efficiencies of samples (1) and (2) and (5) were evaluated by batch experiments for crystal violet and methyl orange dyes. In a typical experiment, 0.01 g of adsorbent was added to each of 25, 50, 100, 150, 200, 250, 300, 350 mg/L dye solution of volume 10 mL. The mixture was shaken at room temperature for 24 h, then the capacity was determined.

The adsorption data was fitted by Langmuir and Freundlich models which relate qe (mg/g) the amount of solute adsorbed by the solid with the remaining concentration of dye in solution Ce (mg/L). The equilibrium data were analyzed using linear and nonlinear isotherms equations of Langmuir and Freundlich models, Table 2 [46,47,48,49]. Modelling and adjustment was performed using Origin program v8.

**Figure 10:**
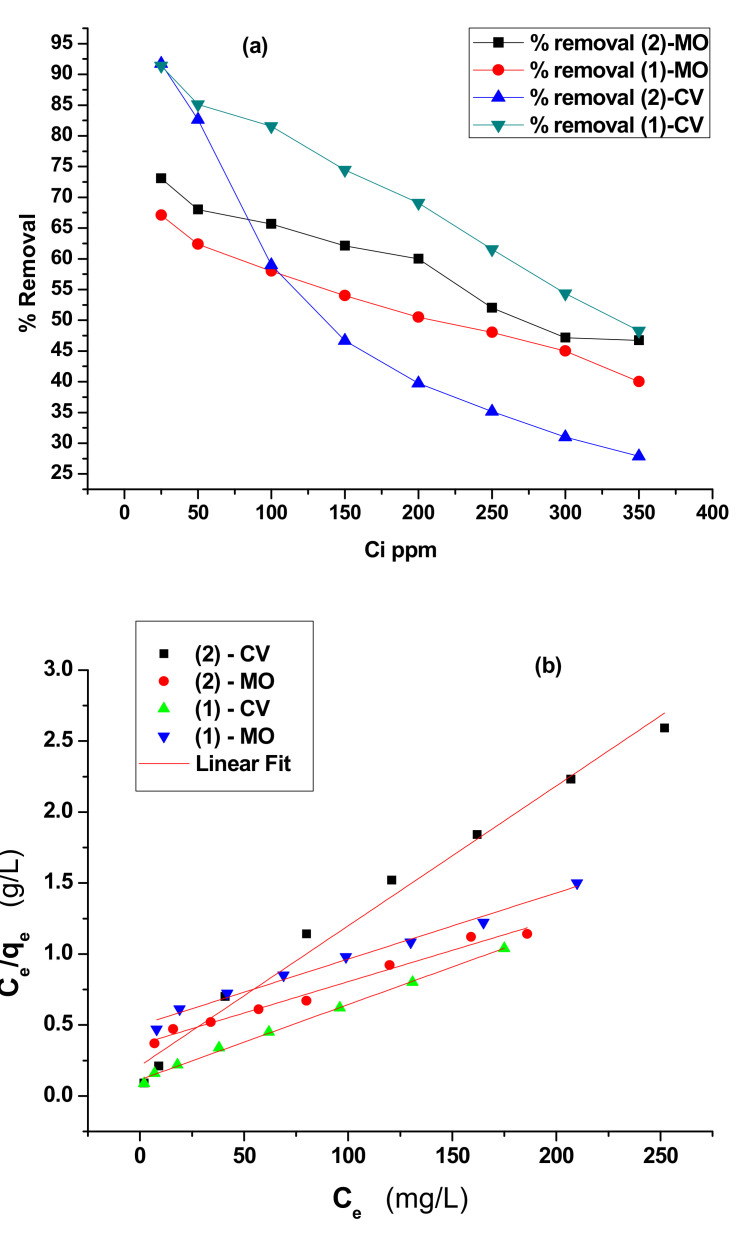
Equilibrium adsorption studies (**a**) Effect of initial concentration and **(b**) Linear fit to Langmuir model for the adsorption of CV and MO dyes by adsorbents (1) and (2) at 25 °C. For 25, 50, 100, 150, 200, 250, 300, 350 mg/L dye initial concentration. Adsorbent mass is 0.01 g and the volume of solution is 10 mL, agitation time 24 h.

**Figure 11:**
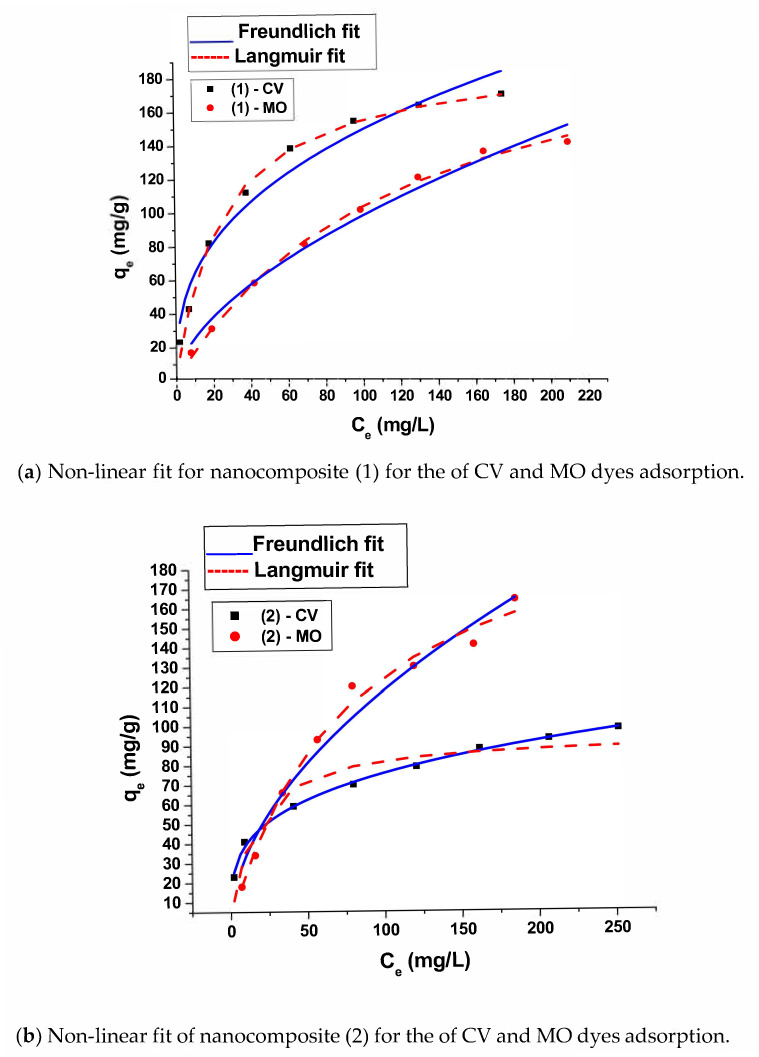
Langmuir and Freundlich models non-linear fit for the adsorption of CV and MO dyes, (**a**) by nanocomposite (1) and (**b**) by nanocomposite (2).

The adsorption data is well fitted with Langmuir model using linear curve fit (Figure 10b) and nonlinear curve fit (Figure 11) indicating the presence of chemical monolayer adsorption onto a surface with a finite number of active sites. Using Langmuir non linear model, the calculated maximum uptake q_max_ of CV and MO is 192.3 and 224.5 (mg/g) for sample (1) respectively. It is for sample (2) 94.1 and 225.5 mg/g respectively. It is obvious that the uptake of MO is greater than that of CV dye by both adsorbents. 

The positive charges built up in adsorbent (2) as a result of protonation of (1) by 10% HCl treatment, caused the capacity to doubly increase when replacing the cationic CV by anionic MO dye. Because the repulsion existing between similar positive charges of the adsorbent and CV dyes was replaced by an attractive force between the positive charges of adsorbents and anionic charges of MO dyes [14,49]. Also, the functional groups containing oxygen and nitrogen atoms present on graphite sheets of nanocomposites (confirmed by XPS) have large negative density can also help in the adsorption of cationic dyes [14,16]. Thus, electrostatic interactions contribute to the adsorption capacity strength [15]. However, ion exchange existing between cationic dyes and protons of (2) can also be responsible for the existing adsorption of cationic CV by (2) [49].

The uptake capacity values of (1) for CV and MO showed little difference, although the dyes are oppositely charged. This indicates that not only charge interaction is responsible for the adsorption process but also π-π interaction existing between aromatic rings of dyes and cobalt nanocarbon adsorbents [14,15,16,35]. 

The higher cobalt species content in (1) compared to (2) can be responsible for the increase in adsorption of CV dye due to increase in complexation of cobalt ions with CV, [14,15,16,49].

The 𝑅_𝐿_ values are also calculated where 𝑅_𝐿_ = 1/(1 + 𝐾_𝐿_𝐶_0_) and 𝐾_𝐿_ (L.mg^−1^) is a coefficient related to the affinity between the sorbent and sorbate. 𝐶_0_ is the maximum initial concentration. The obtained value of 𝑅_𝐿_ for the materials ranges from 0.04 to 0.25. Thus, the values of 𝑅_𝐿_ are between 0 and 1, this indicates that adsorption of dyes is favourable, Table 2. 

Freundlich models were also fitted using linear (Supplementary Figure S6) and nonlinear curve fit (Figure 11) for the adsorption of CV and MO by nanocomposites (1) and (2). The Freundlich isotherm constant *n_F_* indicates the heterogeneity factor. The obtained *n_F_* values are lower than 1 (ranges from 0.3 to 0.7). This reveals the existence of strong adsorption between adsorbent and sorbate, Table 2.

### 3.4. Adsorption Kinetics

Adsorption kinetics study the change of adsorption capacity with respect to time and give information about the nature of adsorption mechanisms. It was found that the capacity increases with time of adsorption for all the dyes by all nanocomposites. The slope of qt versus time is high at initial adsorption period. The high adsorption rate at initial stages is related to the availability of large number of vacant sites which saturate with time [16].

The following models are used:

Pseudo-first order model:

(5) $$\log(q_{e}-q_{t})=logq_{e}-\frac{k_{1}t}{2.303}$$

where *k_1_* (min^−1^) is the rate constant of the pseudo-first order model. It can be measured from the slope of the linear plot of log (q_e_ − q_t_) versus *t* (min). *q_t_* (mg/g) is the amount of adsorption at time *t* (min), and *q_e_* (mg/g) is the amount of adsorption at equilibrium. 

Pseudo-second order model:

(6) $$\frac{t}{q_{t}}=\frac{1}{k_{2}{\left(q_{e}\right)}^{2}}+\frac{t}{q_{e}}$$

where *k_2_* (g/mg.min), the rate constant of the pseudo-second order and *q_e_* can be calculated from the intercept and slope respectively, from the plot of t/q_t_ versus *t*.

**Figure 12:**
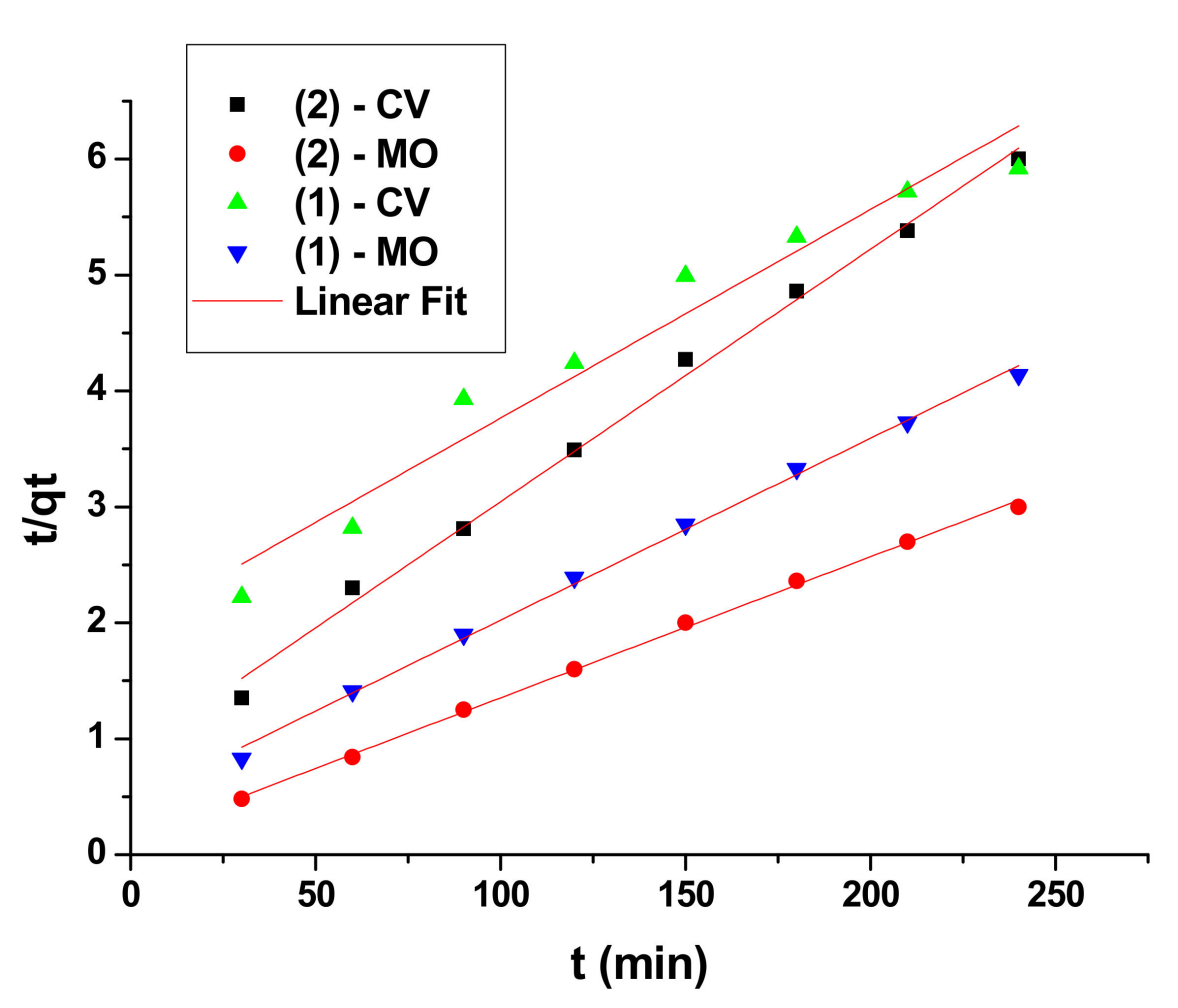
The rate adsorption of nanocomposites (1) and (2) for the CV and MO dyes fitted by pseudo-second-order rate equation. (100 mg/L dye, adsorbent 0.05 g, and 50 mL of total volume solution was used in this experiment).

The experimental kinetic data of nanocomposites (1) and (2) were fitted to both pseudo-first order (Supplementary Figure S7) and pseudo-second-order (Figure 12) models for the adsorption of CV and MO dyes respectively, (Table 3) [50,51]. The values of R^2^ values for the fitting of pseudo second order rate are a little higher than those for pseudo first order rate, except for 1-CV system where the R^2^ value for pseudo first order is higher than that for pseudo second order. It can be concluded that the sorption process follows pseudo second order adsorption rate for most of nanocomposites. Pseudo first order kinetics indicates that adsorption occurs by diffusion through the interface; while pseudo second order assumes that the rate-limiting step is chemical sorption [15,49]. In general, both adsorbents kinetic rate constants are higher for the adsorption of MO than that of CV dyes. This could be related to the fact that the size, and molar mass of MO is smaller than that of CV dyes. 

### 3.5. Adsorption Thermodynamics

**Figure 13:**
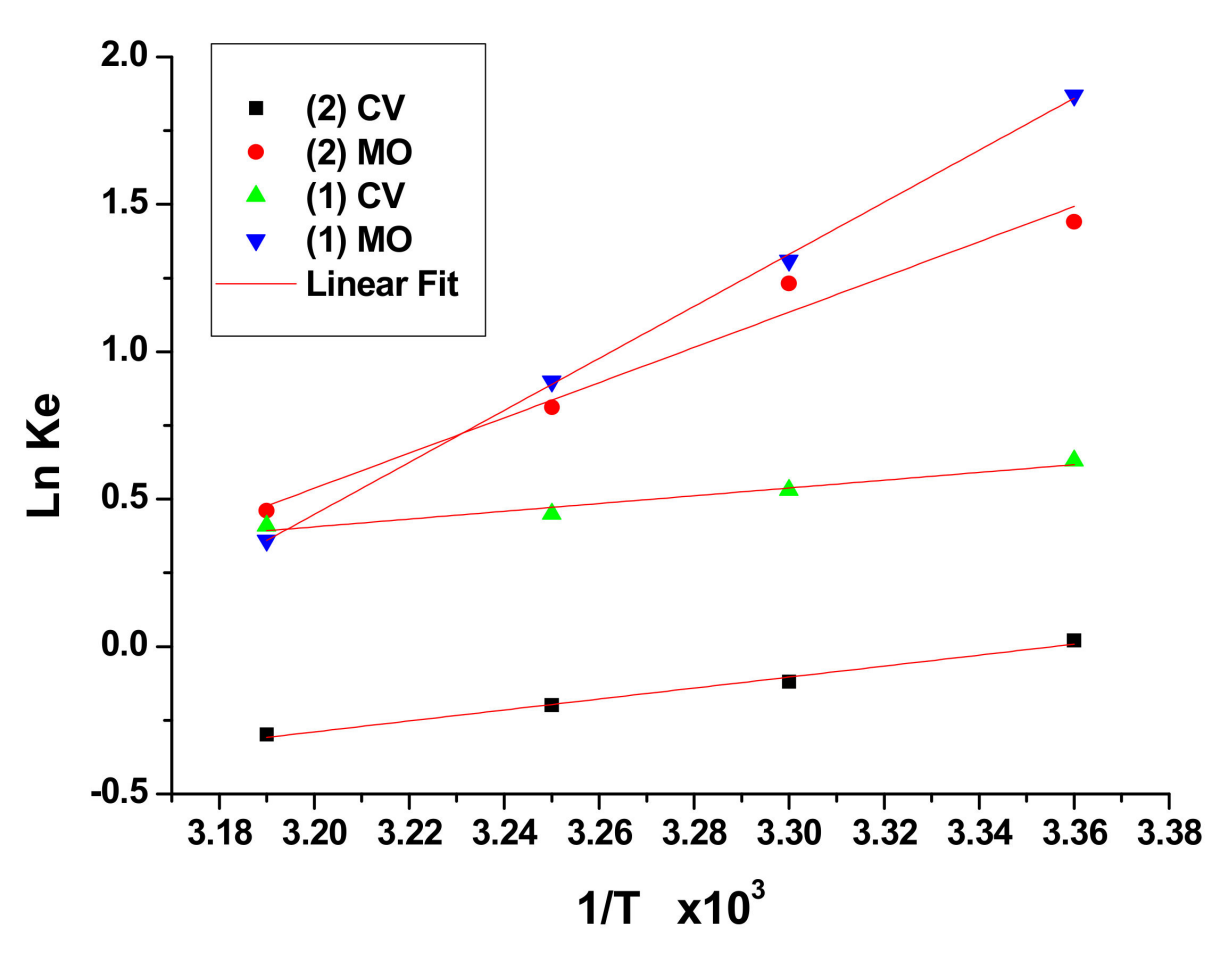
Van’t Hoff plot of Ln Ke vs. 1/T for the adsorption of MO or CV dye (100 mg/L) by 0.01 g of (1) or (2) materials. (Volume of dye solution = 10 mL).

The standard enthalpy change (Δ*H*^◦^) and standard entropy change (Δ*S*^◦^) were calculated by plotting ln *K* versus 1/*T* using Van’t Hoff equation (Figure 13). The temperatures tested are 298, 303, 308 and 313 ^◦^K. Gibbs free energy (*ΔG*^◦^) of adsorption was determined using the following equation (*ΔG*^◦^) = *(ΔH*^◦^)−*T*(Δ*S*^◦^), where *K* is the adsorption equilibrium constants, [52]. Inspection of the results indicates that the adsorption process is exothermic and that the Δ*H*^◦^ values of adsorption increase in the order of (1)-CV (−11.69), (2)-CV (−16.26), (2)-MO (−52.33) and (1)-MO (−77.15) kJ/mol. Moreover, the negative values of the Gibbs free energy for (1) and (2) dyes uptake suggests that the process is spontaneous and it is more spontaneous in the case of MO dyes than CV dyes for both adsorbents. Δ*G^◦^* for adsorption of MO by (1) and (2) are −4.74 and −3.76 respectively. While, it is only −1.53 and −0.17 kJ/mol for the adsorption of CV by (1) and (2) respectively. 

The *ΔG^◦^* values range between −0.17 to −4.74 kJ/mol. This indicates that the adsorption process is mainly physical in nature, since the *ΔG◦* values lie between 0 to −20 kJ/mol [53]. Negative values of *ΔS*^◦^ demonstrate that extraction is dependent on the affinity of adsorbent towards dyes to form more ordered adduct and also on the decrease in the presence of mobile dyes in aqueous solution. The Δ*S^◦^* values for the adsorption of CV and MO are −0.0341, −0.2433 for (1), and −0.054, −0.163 kJ/mol for (2) respectively. 

### 3.6. Adsorption Mechanism

Co-HGC@SiO_2_/HF (3) was prepared in order to understand more the structure of Co-HGC@SiO_2_ (1). In (3) the silica was removed by treatment with HF acid leaving void spaces in the structure, TEM analysis Figure 2c. SEM shows the nanocarbon coat with lost silica matrix, Figure 1d. The void volume was further proved by BET analysis. This confirms successful coating of silica by cobalt graphitic nanocarbon coat that acts as efficient adsorptive surface for the aromatic dyes.

Co-HGC@SiO_2_/CV (4) was isolated from (1) after its complete adsorption of crystal violet. XPS of (4) revealed the increase in carbon and nitrogen content on the surface of nanomaterials, and thus prove the successful adsorption of crystal violet dye by (1) and that chemical adsorption can be involved. 

Adsorbent Co-HGC (5) was prepared without silica. Its SEM image indicates exposed cobalt nanoparticles embedded in graphitic carbon (Supplementary Figure S1). [34,35]. This structure facilitates interaction of cobalt species with the dye. Cobalt nanoparticles are expected to enhance adsorption thru binding with electron donors group present in the dyes.

Adsorbent (2) was obtained by HCl acid treatment of (1). Thus (2) acquired extra positive charges due to protonation of nitrogen, sulphur and oxygen groups present on the surface of graphitic carbon. The low CV dye capacity 94.1 mg/g for (2) compared to 192.3 mg/g for (1), can be explained by electrostatic similar positive charge repulsions occurring when the cationic CV dye is adsorbed on (2). The adsorption can be due to π-π interaction between aromatic rings of CV and aromatic graphite matrix of nanocomposites, [35]. In addition, CV can form hydrogen bonds with oxygen and nitrogen groups present on the graphite matrix. Finally, the low capacity of (2) is because it has fewer cobalt species which can bind to electronegative groups of CV [47,48,49,50,51].

In the case of anionic MO uptake, it is expected that (2) will have higher capacity than (1) because of electrostatic attraction that can occur between negatively charged MO and (2) which is rich in protonated groups. However, the capacity is almost similar because of the loss of cobalt species in (2) which complexes with MO [47,48,49,50,51]. 

The adsorption capacity of (5) is similar to (1) for uptake of CV (192 mg/g), while it becomes 769.2 mg/g with anionic dye MO. This can be explained by favourable complexation between cobalt species and the electron donor groups of MO dye. 

### 3.7. Comparison of Adsorption Capacity of Nanocomposites (1), (2) and (5) with Other Adsorbents

New cobalt hierarchical graphitic carbon nanostructure Co-HGC (5) was prepared following the same procedure and conditions as of Co-HGC@SiO_2_ (1). It involves pyrolysis at 850 °C of cobalt phenanthroline sulphate complex with pyrene (1:1) but without the addition of silica. The SEM image of (5) (Supplementary Figure S1) is similar to SEM image of (1) Figure 1a indicating that (1) and (5) have similar surface morphology, as expected since silica is underneath the cobalt nanocarbon coating in (1) and not exposed. As for complete characterization of (5) was out of the scope of this paper. Thus, the adsorption abilities of (5) for CV and MO were studied. Freundlich isotherm model for uptake of MO and CV by (5) (Supplementary Figure S8) was found more suitable than Langmuir model. The Langmuir adsorption capacity of (5) is equal to 769.2 mg/g for MO and 192.3 mg/g for CV. Thus, the adsorption capacity values qmax which reflect the adsorption efficiency follow the following order for MO: (5) > (1) = (2) and for CV: (5) = (1) > (2). This indicates that the use of silica support increases the yield of product with no loss of adsorption efficiency in the case of uptake of CV dye. While it causes a decrease in the capacity in the case of MO dye.

The adsorption capacity of newly prepared hierarchical graphitic carbon cobalt nanostructures with and without silica Co-HGC@SiO_2_ (1) and Co-HGC@SiO_2_/HCl (2) and Co-HGC (5) were compared with other related adsorbents such as nanocomposites, mesoporous carbons, carbon nanotubes, cobalt oxide nanoparticles, zeolites and metal carbon composites, Table 4, [54,55,56,57,58,59,60,61,62,63]. It was found that the nanocomposites (1) and (2) have capacity values close to other adsorbents for uptake of MO and CV dyes. However, (5) is among the adsorbents which have highest capacities. 

### 3.8. Column Adsorption Studies

Continuous flow studies were investigated in order to estimate the efficacies of dyes remediation using Co-HGC@SiO_2_ (1). For this, column adsorption experiments were achieved using a glass Pyrex column loaded with 0.5 g of nanocomposite (1). The column was eluted with MO or CV dyes solution (100 mg/L) at a flow rate of 1.0 mL/min. A breakthrough graph was obtained by plotting C_ads_ of lead (mg/L) versus time t (min) [64,65]. The maximum volume of eluted MO solution that causes exhaustion of column was found to be around 400 mL for the first cycle and 150 mL for the second cycle. While the maximum eluted volume of CV that exhausted the column was around 500 mL for the first cycle and 300 mL for each of the second and third cycles respectively. The column packed with (1) after exhaustion with CV or MO dyes was regenerated with 0.1 M HCl at a flow rate of 1 mL/min. The column was further washed with distilled water till neutrality of eluted solution was achieved. Then, the next cycle was repeated. The column did not suffer clogging. Also, no degradation of adsorbent was observed.

**Figure 14:**
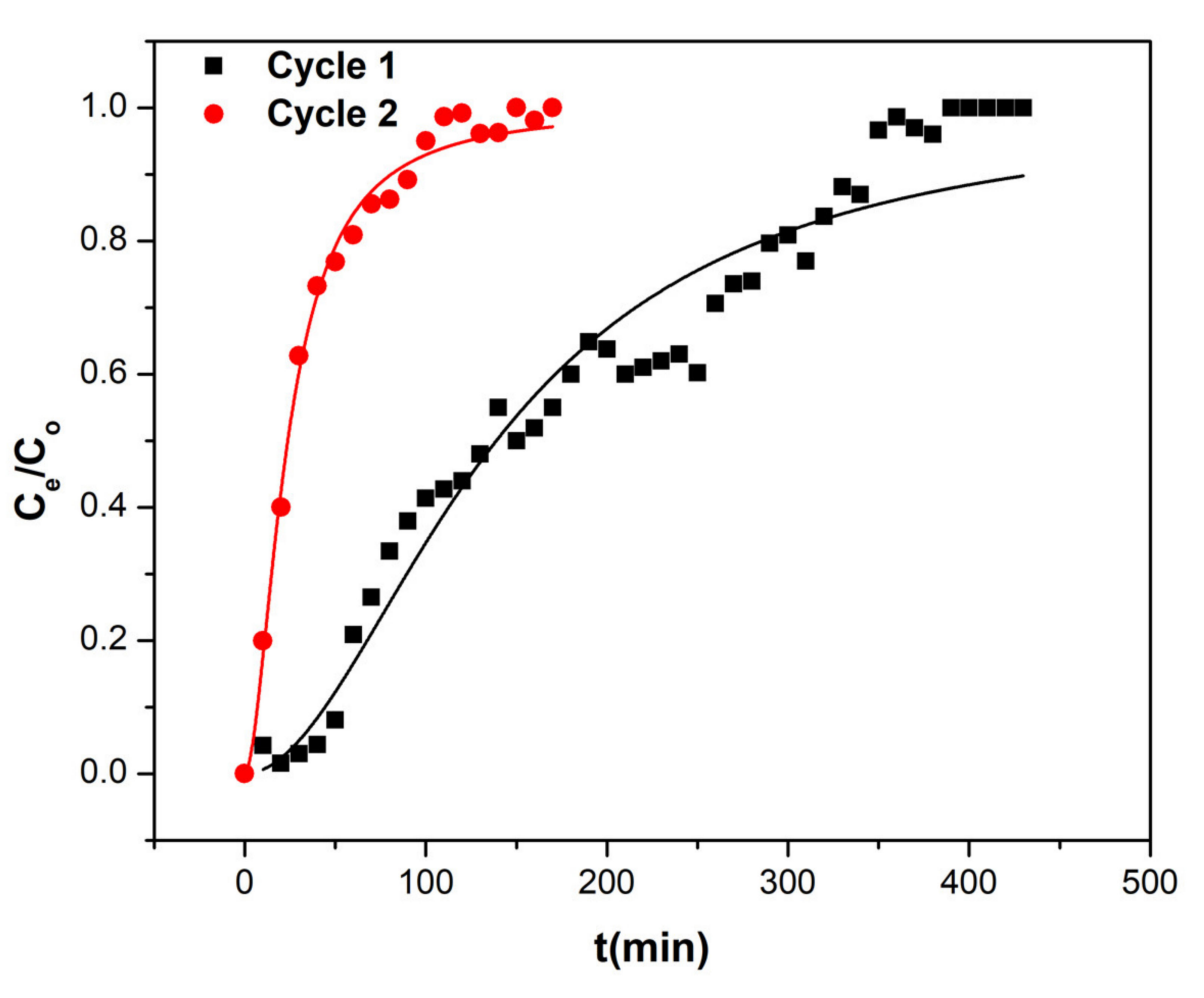
Nonlinear fit of Yan et al. model for adsorption of MO by (1). (0.5 g of (1) packed in column, 100 mg/L of MO dye, 1 mL/min flow rate).

**Figure 15:**
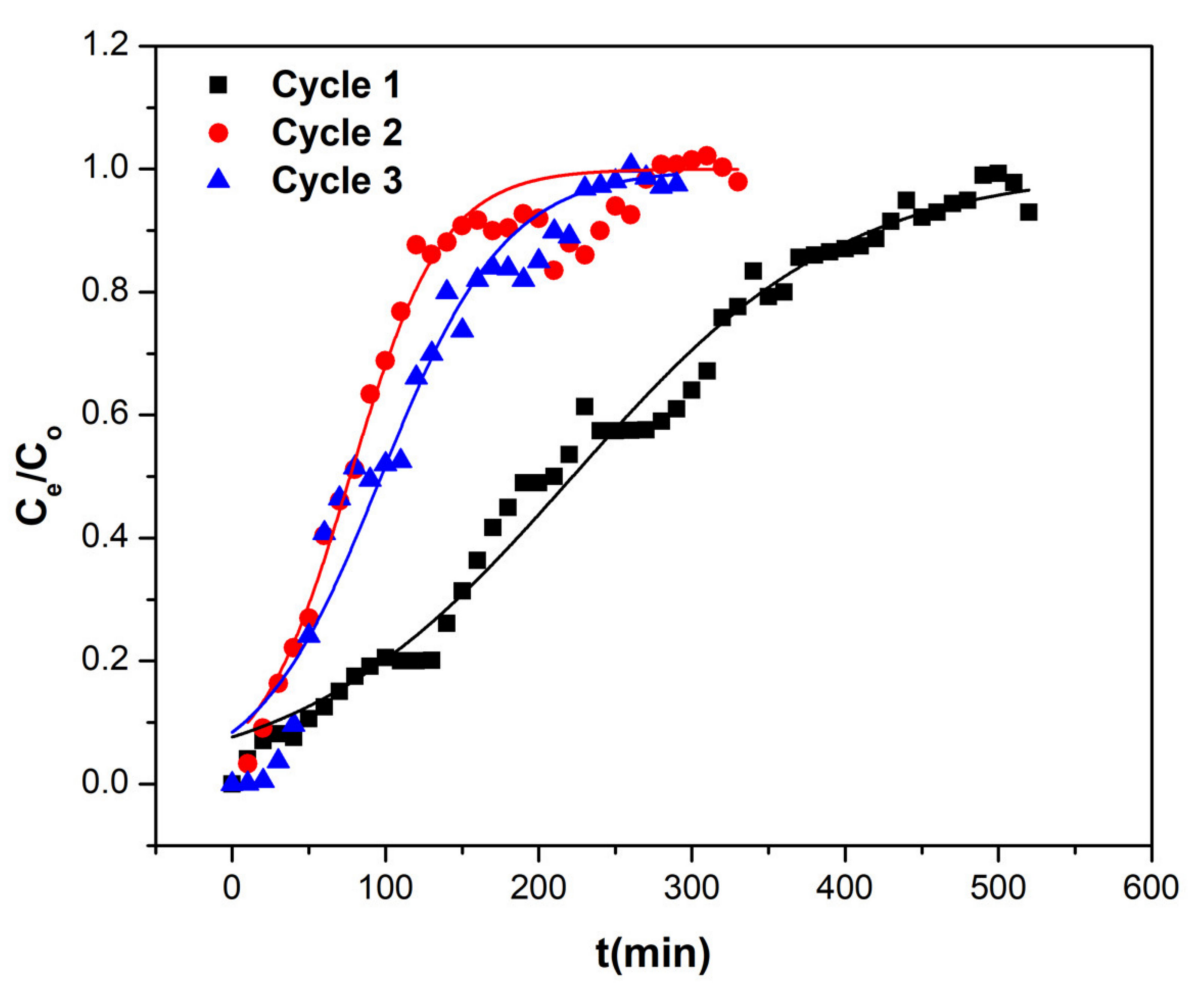
Nonlinear fit of Thomas model of CV by (1). (0.5 g of (1), 100 mg/L of CV dye, 1 mL/min flow rate).

Thomas, Yoon-Nelson, and Yan et al., models were applied using nonlinear regression statistical curve fitting for calculating the maximum uptake capacity of the dyes q (mg/g) and the rate constant k [14,66,67]. The model equations and the calculated parameters are presented in Table 5a for the adsorption of CV dye, and Table 5b for the adsorption of MO dye [68]. It was observed that Yan et al. model (Figure 14 and Supplementary Figure S9) is more suitable than Thomas model (Figure 15 and Supplementary Figure S10) and Yoon-Nelson models (except for the first cycle with CV dye), as evidenced by the higher R^2^ and smaller χ^2^ error function values, Table 5a,b. Application of Yoon-Nelson kinetic model indicated that the half-life of adsorbate breakthrough (τ) is equal to 222 min for the first cycle with CV. It is greater than for the first cycle with MO (163 min) [69]. The fitting of the experimental breakthrough curve with Thomas model gave the values of maximum capacity q_T_ for CV [70]. q_T_ for CV was equal to 44.4, 15.4 and 19.4 mg/g for the first, second and third cycles respectively (Table 5a). In the case of MO adsorption, the capacity q_T_ was found equal to 32.6 and 5.7 mg/g (Table 5b). The obtained results of continuous flow studies depicted that (1) could be used effectively for remediation of cationic and anionic dye from the aqueous phase [70].

## 4. Conclusions

The present work showed a promising synthetic method for preparing cobalt graphitic carbon silica nanocomposites using a simple pyrolytic method, in large scales from the starting materials silica, anthracene and cobalt 1,10-phenanthroline sulfate metal complex. The prepared nanocomposites Co-HGC@SiO_2_ (1) were further modified by the addition of HCl to give (2) or by the addition of HF acid to produce (3). EDX indicates loss of cobalt in the case (2) and loss of cobalt and silica in the case of (3). XPS of nanocomposite (4) obtained after treatment of (1) with crystal violet proved successful adsorption of CV. The nanocomposites have rough porous carbon silica microstructures decorated with cobalt nanoparticles of diameter 50 to 200 nm. TEM indicates that the nanoparticles are made up of graphitic shell and cobalt cores. Nanocomposite (3) has the highest BET surface (217.5 m^2^g^−1^). Cobalt mainly exists as Co(0) and Co(II) as supported by powder X-ray diffraction, XPS. SEM-EDAX. TEM-HAADF imaging provide information about the elemental zone distribution of Si, Co, C, N and S. 

The maximum adsorption capacity of (1) and (2) ranges from 94 to 225 mg/g for MO and CV dyes. Co-HGC (5) prepared without addition of silica has a capacity for CV equal to 192 mg/g, while it is 769.2 mg/g with MO. Nanocomposite (5) shows high capacity compared to the ones reported in the literature. The kinetic adsorption data were fitted with pseudo-second order model. Electrostatic and π–π interactions as well as ion exchange and complexation with cobalt species are responsible for the adsorption process of both dyes with the prepared nanocomposites.

The fixed-bed assays revealed that the column packed with (1) could treat up to 500 mL of dyes (100 mg/L) with a flow rate of 1ml/min. Yan et al. was the best suitable model to describe the dynamic curves. Maximum uptake in continuous studies was found as 32.6 mg/g with MO and 44.4 mg/g with CV dye. The column can be recycled three times. Therefore, Co-HGC@SiO_2_ can be used as an efficient and fast adsorbent to treat effluents containing cationic and anionic dyes.

## Figures and Tables

**Table 1:** EDX for the nanocomposites (1), (2) and (3).

	(1)		(2)		(3)	
Element	Weight%	Atomic%	Weight%	Atomic%	Weight%	Atomic%
C	36.38	56.77	34.28	46.51	78.28	85.65
O	20.39	23.88	37.29	37.99	12.08	9.91
Si	11.41	7.61	24.48	14.21	9.18	4.29
S	6.06	3.54	0.93	0.47	0.25	0.10
Co	25.76	8.19	2.03	0.56	0.21	0.05

**Table 2:** Langmuir and Freundlich isotherm obtained using linear and (nonlinear equations) at 25 °C, for the adsorption of CV and MO dyes for nanocomposites (1) or (2). The values in brackets are for the results of nonliear model fits.

	(1) CV	(2) CV	(1) MO	(2) MO
**Langmuir**				
q_max_ (mg/g)	188.68 (192.34)	101.4 (94.14)	214.59 (224.47)	226.76 (225.48)
*R_L_*	0.0664 (0.0651)	0.0584 (0.0415)	0.234 (0.252)	0.191 (0.192)
*R^2^*	0.9970 (0.9955)	0.9862 (0.9800)	0.9872 (0.9967)	0.9841 (0.9889)
*SD* (χ^2^)	0.0198 (16.39)	0.1160 (82.67)	0.0413 (8.33)	0.0404 (35.57)
**Freundlich**				
*K_F_* (mg/g)(L/mg)^1/n^	18.54 (27.13)	19.90 (20.60)	4.44 (6.67)	5.55 (9.65)
*n_F_*	0.460 (0.369)	0.290 (0.282)	0.670 (0.580)	0.666 (0.540)
*R^2^*	0.9734 (0.9630)	0.9936 (0.9969)	0.9918 (0.9837)	0.9761 (0.9697)
*SD* (χ^2^)	0.1280 (134.68)	0.0431 (2.49)	0.0741 (42.37)	0.1301 (96.93)

**Table 3:** Parameter values and correlation coefficients for pseudo-first-order and pseudo-second order kinetics models for adsorption of CV and MO dyes by (1) or (2).

	Pseudo First Order Kinetics				Pseudo Second Order Kinetics				
	1 CV	1 MO	2 CV	2 MO		1 CV	1 MO	2 CV	2 MO
*qe* (mg/g)	55.03	38.2	38.37	21.64	*qe* (mg/g)	55.56	63.94	45.87	81.97
k_1_ (min^−1^)	3.38 × 10^−3^	4.26 × 10^−3^	3.11 × 10^−3^	2.65 × 10^−3^	k_2_ (g mg^−1^ min^−1^)	1.65 × 10^−4^	5.37× 10^−4^	4.91 × 10^−4^	1.11 × 10^−3^
*R*	0.9934	0.9898	0.9758	0.8768	*R*	0.9794	0.9987	0.9977	0.9993
*R^2^*	0.9868	0.9797	0.9522	0.7688	*R^2^*	0.9592	0.9974	0.9954	0.9986
*SD*	0.0081	0.2118	0.0241	0.0502	*SD*	0.2942	0.0637	0.1167	0.0351

**Table 4:** Comparison of adsorption capacity of the new adsorbents (1), (2) and (5) for uptake of CV and MO dyes with other adsorbents.

Dye	Adsorbent	Capacity mg/g	Reference
MO	MOF-derived nanoporous carbon	872.0	[54]
	Mesoporous ZSM-5 zeolite	25.0	[55]
	p-CNTs/chlorine chloride	310.2	[56]
	Amorphous CNTs	21.5	[57]
	Reduced graphene oxide	243.9	[58]
	Co_3_O_4_ NPs	46.1	[59]
	Co-HGC@SiO_2_ (1)	224.5	This study
	Co-HGC@SiO_2_/HCl (2)	225.5	This study
	Co-HGC (5)	769.2	This study
CV	Graphene oxide/activated carbon	70.0	[60]
	MWCNTs-OH	988.0	[61]
	Fe_3_O_4_/graphene/ biochar composite	436.7	[62]
	Zirconia/carbon composite	243.0	[63]
	Co-HGC@SiO_2_ (1)	192.3	This study
	Co-HGC@SiO_2_/HCl (2)	94.1	This study
	Co-HGC (5)	192.2	This study

**Table 5:** Column kinetic models, equations and parameters for the adsorption of CV by (1). (0.5 g of (1) packed in column, (a) by 100 mg/L of CV dye, (b) by 100 mg/L of MO dye; Flow rate = 1 mL/min.

	(a) CV Dye			(b) MO Dye	
	Cycle 1	Cycle 2	Cycle 3	Cycle 1	Cycle 2
	**Thomas** $\frac{C_{e}}{C_{o}}=\frac{1}{1+e^{\frac{K_{T}}{Q}(q_{T}m-C_{o}V)}}$				
χ^2^	0.00151	0.0033	0.0049	0.0055	0.00441
SSE	0.0773	0.1023	0.1372	0.2253	0.0706
R^2^	0.9851	0.9631	0.9576	0.9390	0.9486
k_T_ (mL/min.mg)	0.112	0.3293	0.246	0.12	0.607
q_T_ (mg/g)	44.42	15.36	19.40	32.62	5.73
	**Yoon-Nelson** $\frac{C_{e}}{C_{o}}=\frac{1}{1+e^{K_{YN}(\tau -t)}}$				
χ^2^	0.00151	0.0033	0.0049	0.0055	0.00441
SSE	0.0772	0.1022	0.1373	0.2254	0.0706
R^2^	0.9851	0.9631	0.9576	0.9390	0.9486
k_YN_ (min ^−1^)	0.0112	0.0329	0.0246	0.0120	0.0607
q_YN_ (mg/g)	44.42	15.36	19.40	32.62	5.73
_Ʈ (min)_	222.09	76.81	97.02	163.13	28.65
	**Yan** $\frac{C_{e}}{C_{o}}=1-\frac{1}{1+{\left(\frac{Q^{2}t}{K_{Y}q_{Y}m}\right)}^{\frac{K_{Y}C_{o}}{Q}}}$				
χ^2^	0.00305	0.00201	0.00263	0.00545	7.82 × 10^−4^
SSE	0.15553	0.06236	0.07374	0.22335	0.01252
R^2^	0.9700	0.9770	0.9772	0.9396	0.9909
k_Y_ (mL/min.mg)	0.0247	0.0246	0.0233	0.0192	0.0179
qy (mg/g)	16.38	5.73	7.34	14.45	2.65

## Data Availability

Not applicable.

## Supplementary Materials

The following are available online, Figure S1: SEM image of Co-HGC (5), Figure S2: EDX spectrum of Co-HGC@SiO2 (1) and Co-HGC@SiO2/HCl (2), Figure S3: XPS survey spectra of nanocomposites (1), (2) and (4), Figure S4: XPS deconvoluted spectra of cobalt 2p for nanocomposites (1), (2) and (4), Figure S5: The O1s XPS deconvoluted spectrum of nanocomposites (1), (2) and (4), Figure S6: Linear fit to Freundlich model for the adsorption of CV and MO dyes by adsorbents (1) and (2) at 25 °C, Figure S7: Pseudo-first-order rate plot for the adsorption of CV and MO dyes by (1) and (2) at 25 °C, Figure S8: Freundlich isotherm linear model for uptake of MO and CV dye by nanocomposites (5), Figure S9: Nonlinear fit of Yan et al. model for adsorption of CV by (1), Figure S10: Nonlinear fit of Thomas model for adsorption of MO by (1), Table S1: Zeta potential of the all three nanocomposites (1), (2) and (3) as a function of pH.

## Sample Availability

Samples of the compounds are available from the authors.
