# Supplementary material for: Cobalt–Carbon Nanoparticles with Silica Support for Uptake of Cationic and Anionic Dyes from Polluted Water
Source: Molecules. 2021 Dec 10;26(24):7489. doi: 10.3390/molecules26247489 (PMC8704004; doi:10.3390/molecules26247489)
Supplement: Supplementary file 1 [file molecules-26-07489-s001.zip › molecules-1466752-supplementary.pdf]

Supplementary documents

Molecules ID: 1466752

Cobalt–carbon nanoparticles with silica support for uptake of cationic and anionic dye from polluted water

Hassan H. Hammud,[1]* Ranjith Kumar Karnati,[1] Nusaybah Alotaibi,[1] Syed Ghazanfar Hussain,[2] Thirumurugan Prakasam[3]

[1]Department of Chemistry, College of Science, King Faisal University, P.O Box 400 Al-Ahsa 31982, Saudi Arabia.

[2]Department of Physics, College of Science, King Faisal University, P.O Box 400 Al-Ahsa 31982, Saudi Arabia.

[3]Chemistry Program, New York University Abu Dhabi (NYUAD), Abu Dhabi United Arab Emirates.

** E-mail: hhammoud@kfu.edu.sa*

Supplementary Table S1. Zeta potential of the all three nanocomposites (1), (2) and (3) as a function of pH.

Nanocomposite	E mV (pH=2)	E mV (pH=4)	E mV (pH=7)	E mV (pH=10)	E mV (pH=14)
(1)	36.2	17.4	-1.6	-3.6	-11.4
(2)	19.8	9.6	4.9	-1.2	-18.2
(3)	9.2	3.9	-12.6	-18.6	-26.3

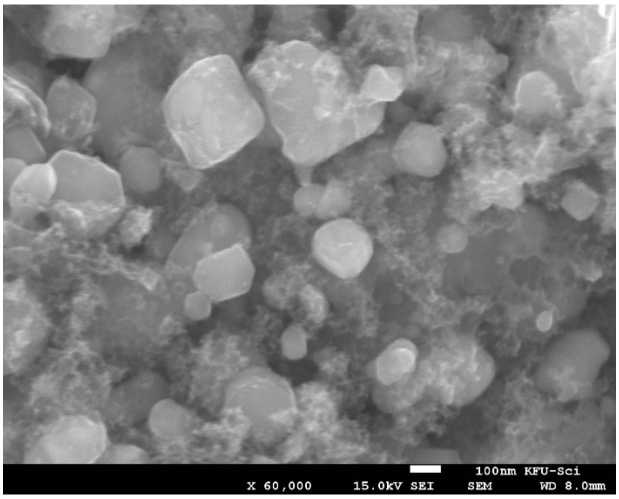

Supplementary Figure S1. SEM image of Co-HGC (5).

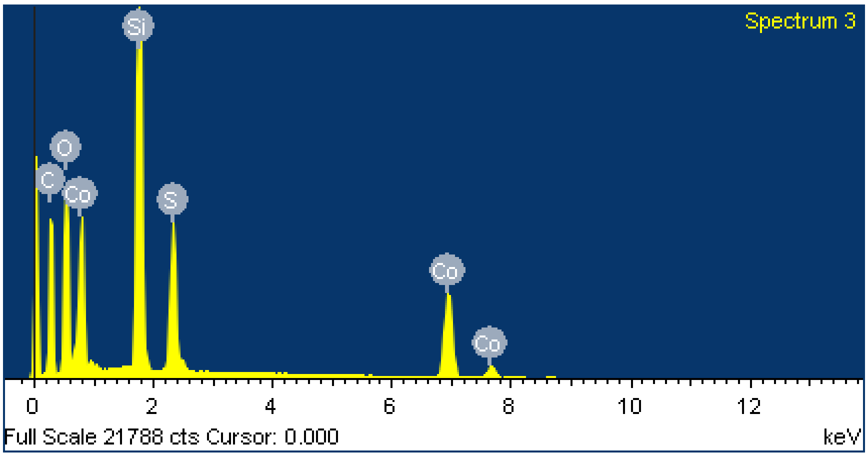

a) EDX of (**1**)

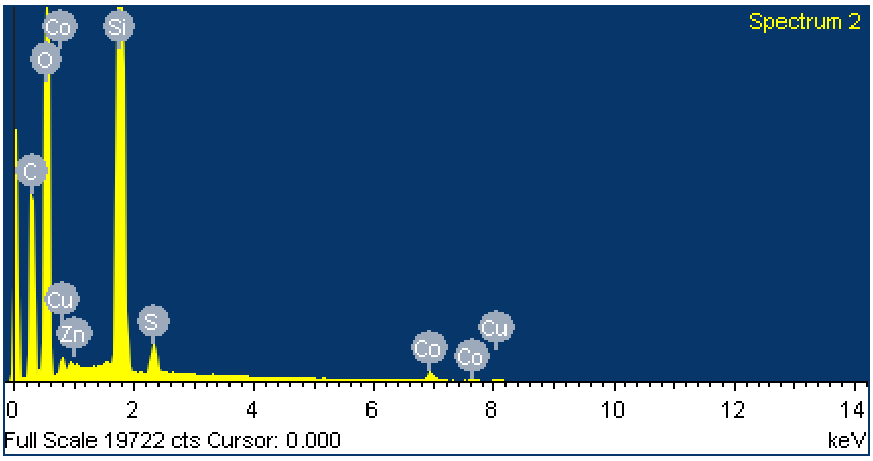

b) EDX of (**2**)

SI. Figure S2. EDX spectrum of Co-HGC@SiO_2 (1) and Co-HGC@SiO_2 /HCl (2)

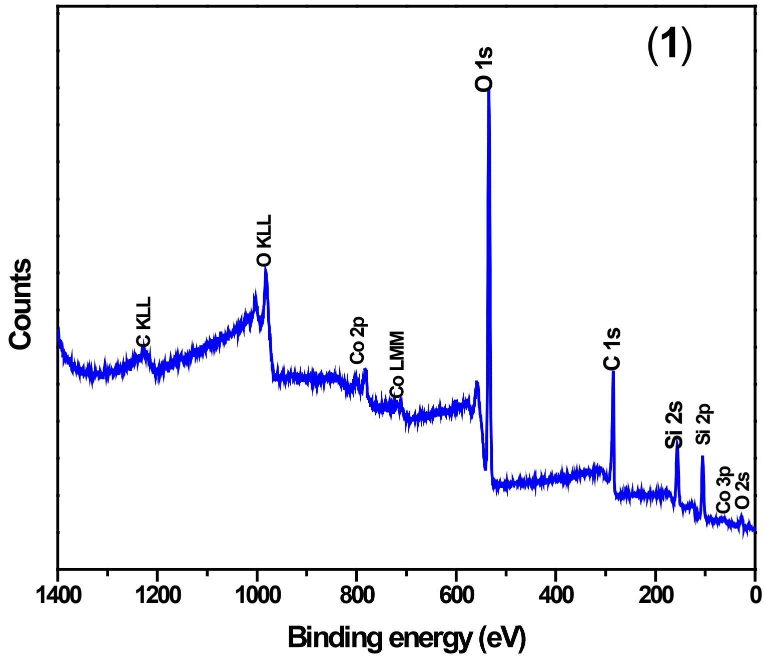

a) XPS survey spectra of (1)

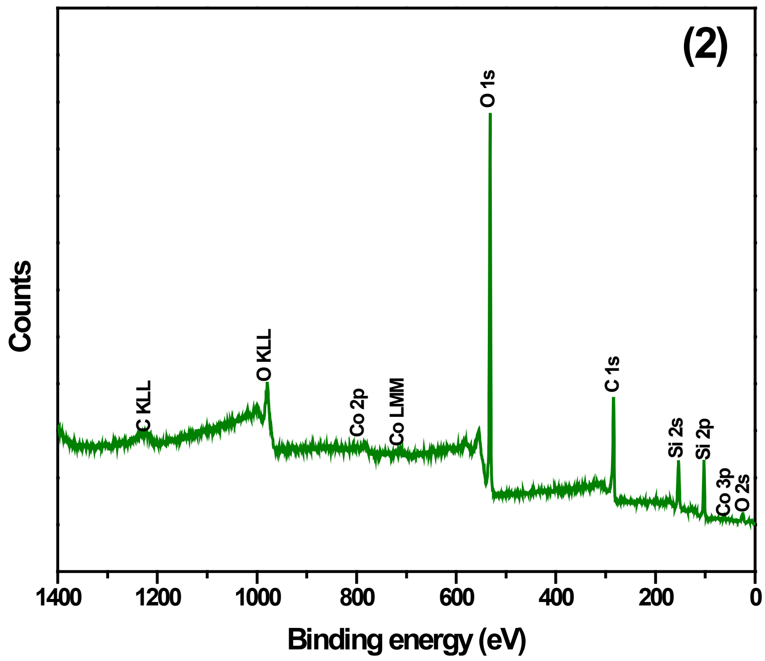

b) XPS survey spectra of (2)

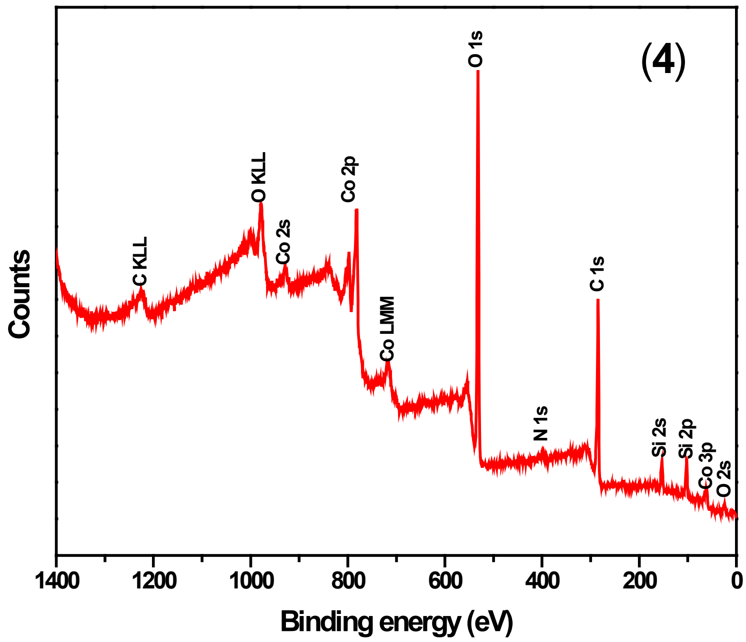

c) XPS survey spectra of (4)

Supplementary Figure S3. XPS survey spectra of nanocomposites (1), (2) and (4).

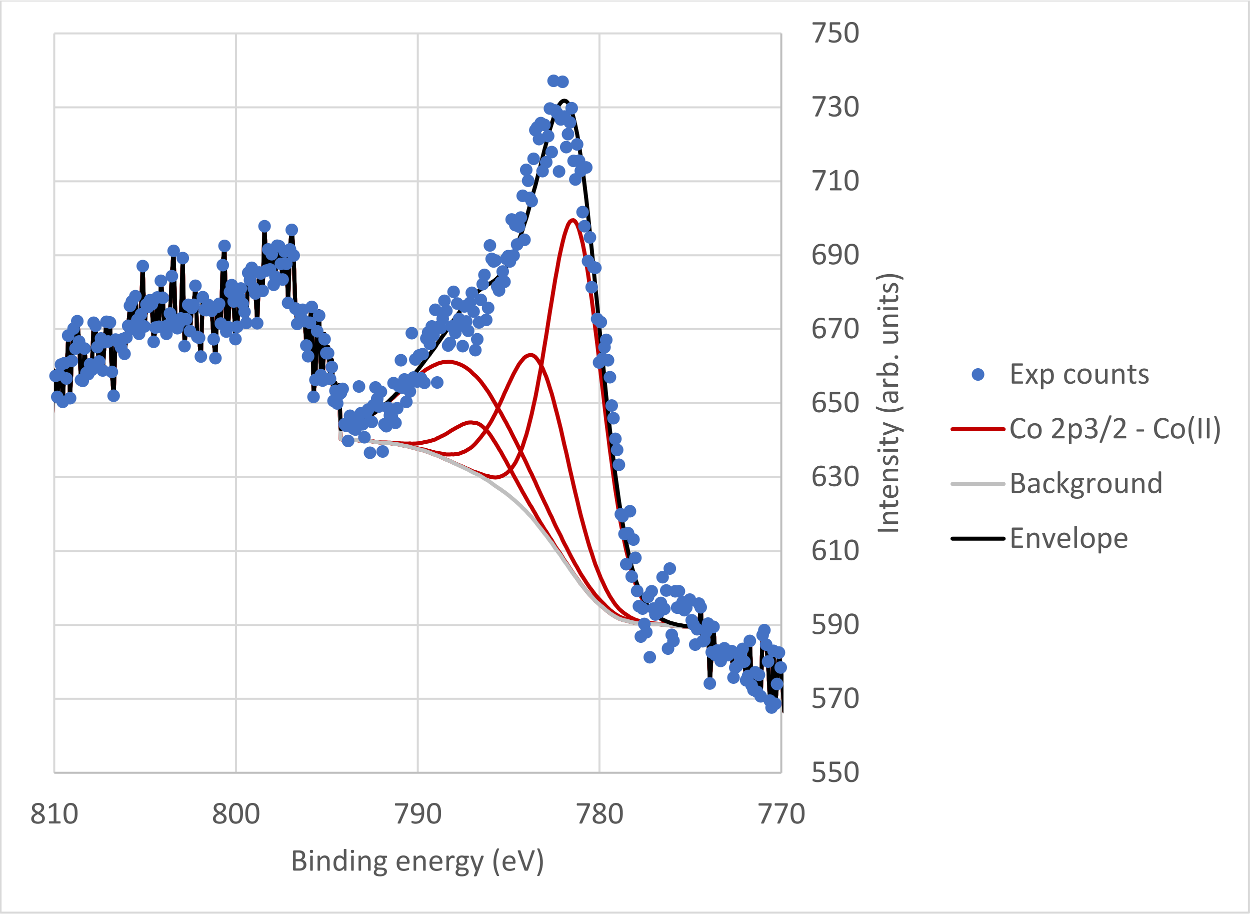

a) XPS deconvoluted spectra of cobalt 2p for (1)

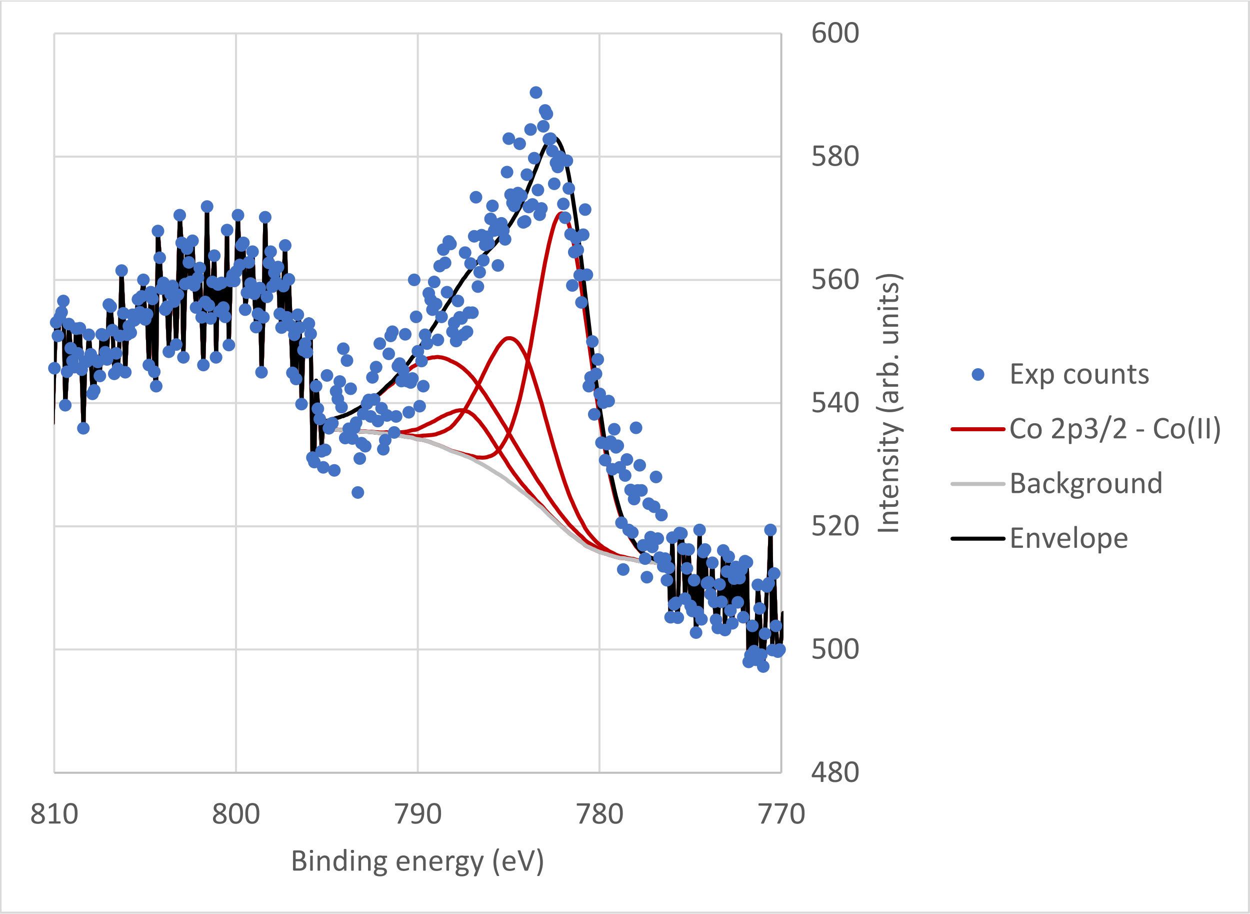

b) XPS deconvoluted spectra of cobalt 2p for (2)

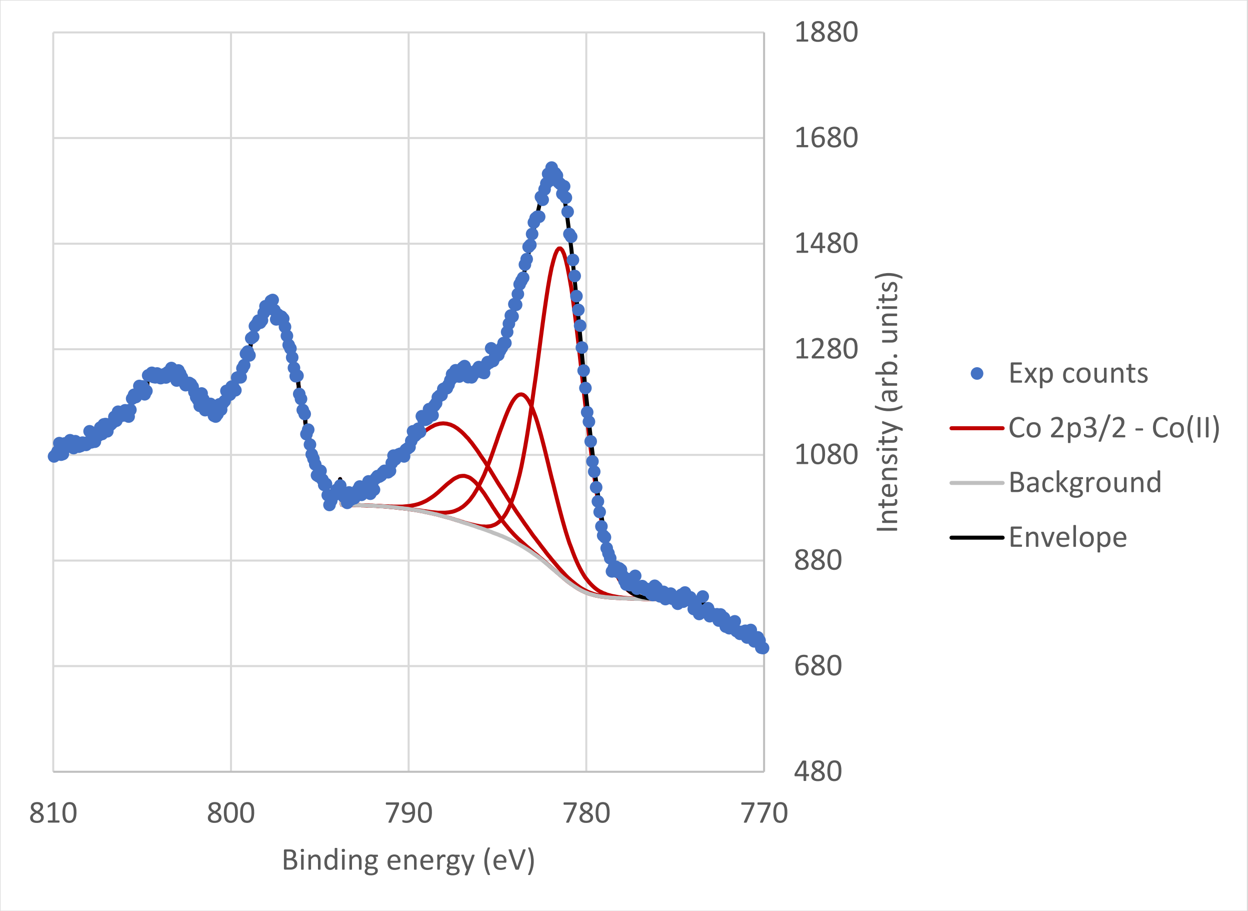

c) XPS deconvoluted spectra of cobalt 2p for (4)

Supplementary Figure S4. XPS deconvoluted spectra of cobalt 2p for nanocomposites (1), (2) and (4)

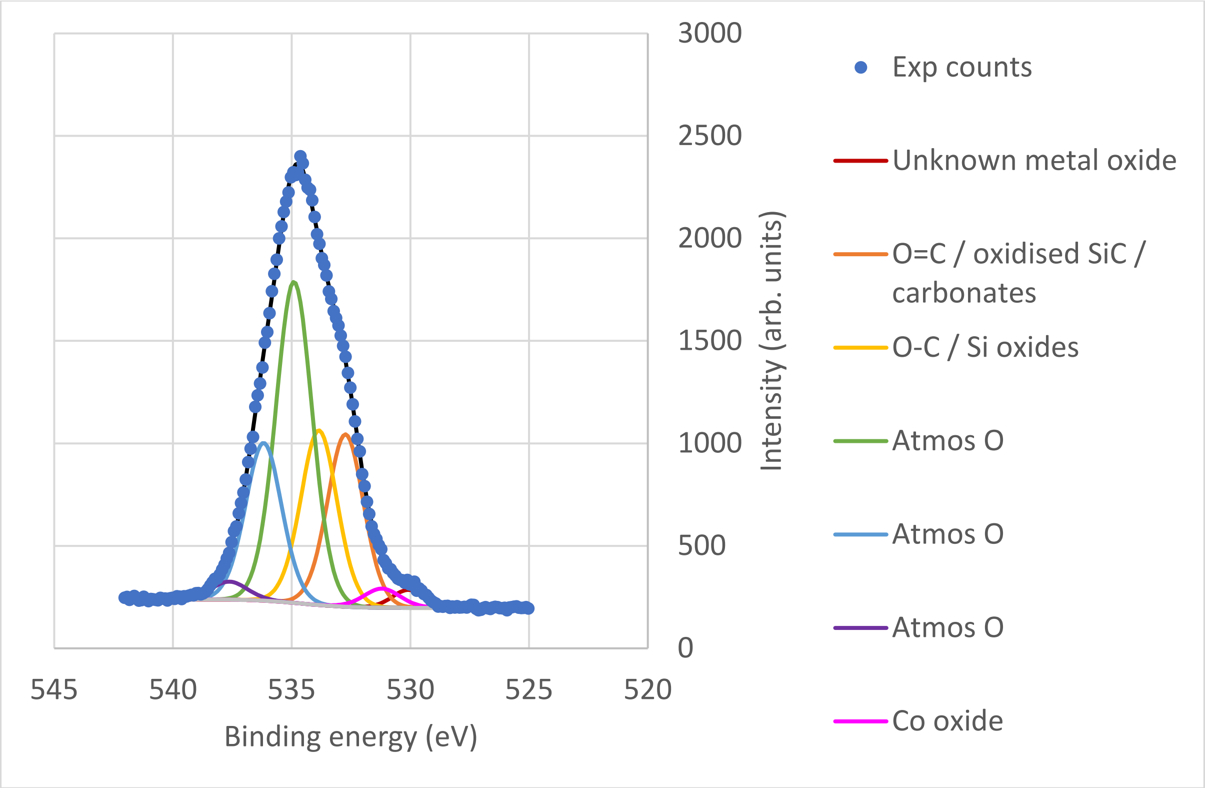

a) The O1s XPS deconvoluted spectrum of (1)

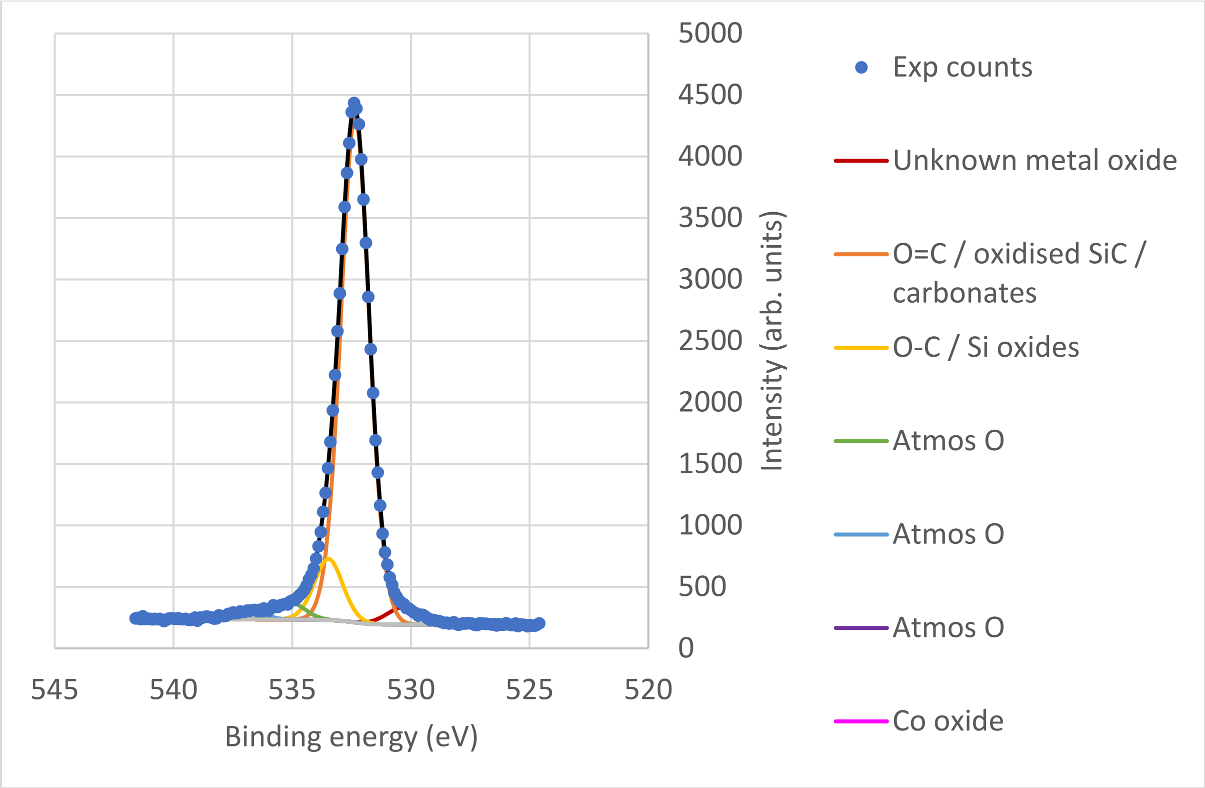

b) The O1s XPS deconvoluted spectrum of (2)

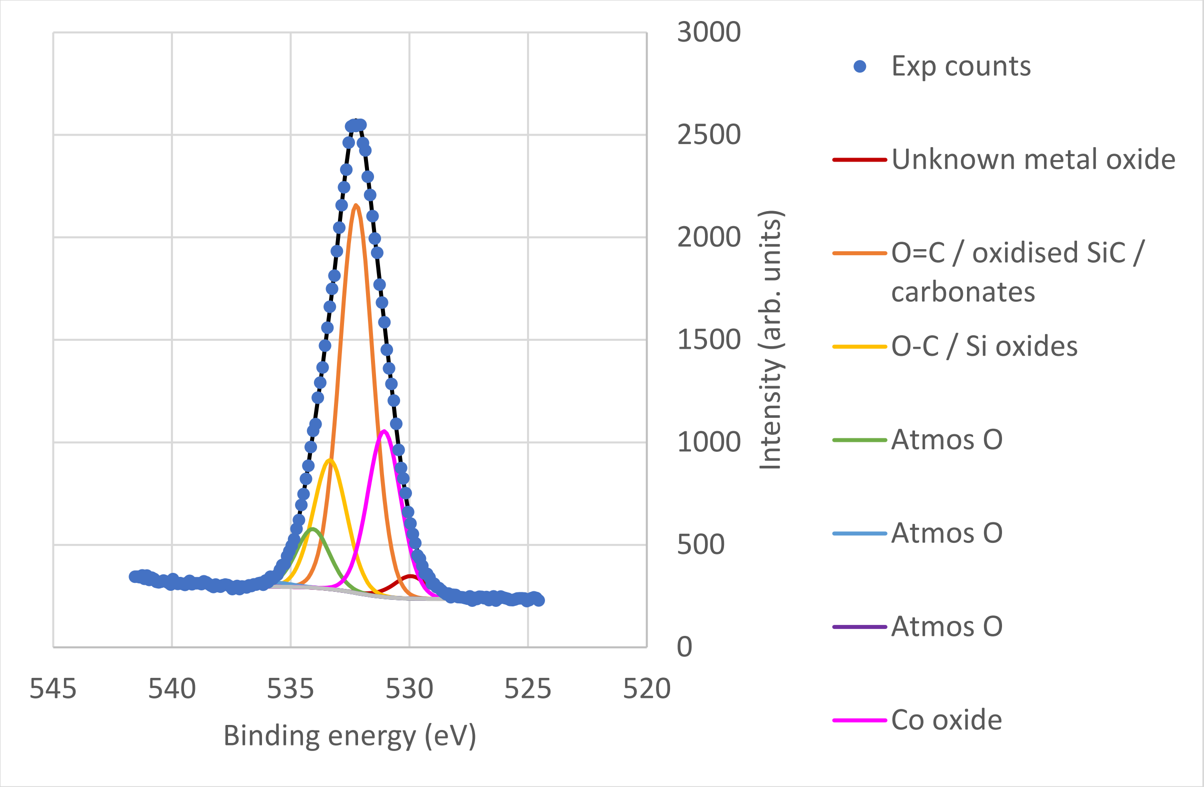

c) The O1s XPS deconvoluted spectrum of (4)

Supplementary Figure S5. The O1s XPS deconvoluted spectrum of nanocomposites (1), (2) and (4).

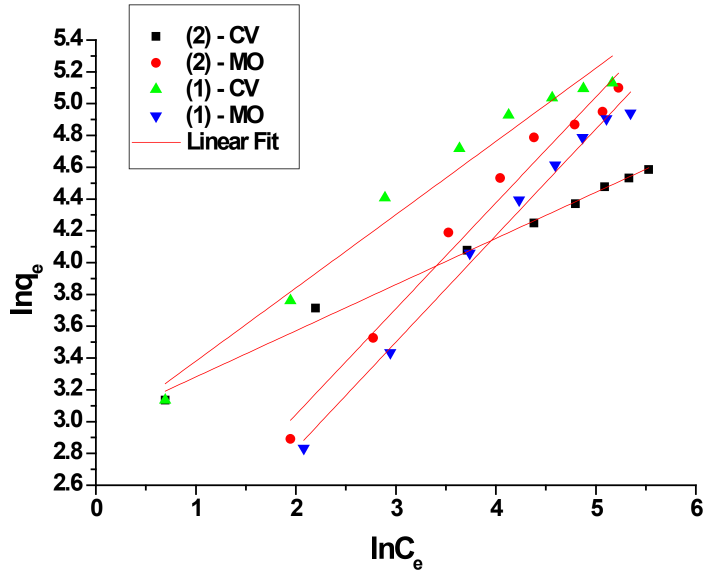

Supplementary Figure S6. Linear fit to Freundlich model for the adsorption of CV and MO dyes by adsorbents (1) and (2) at 25 °C. For 25, 50, 100, 150, 200, 250, 300, 350 ppm dye. Adsorbent mass is 0.01 g and the volume of solution is 10 mL.

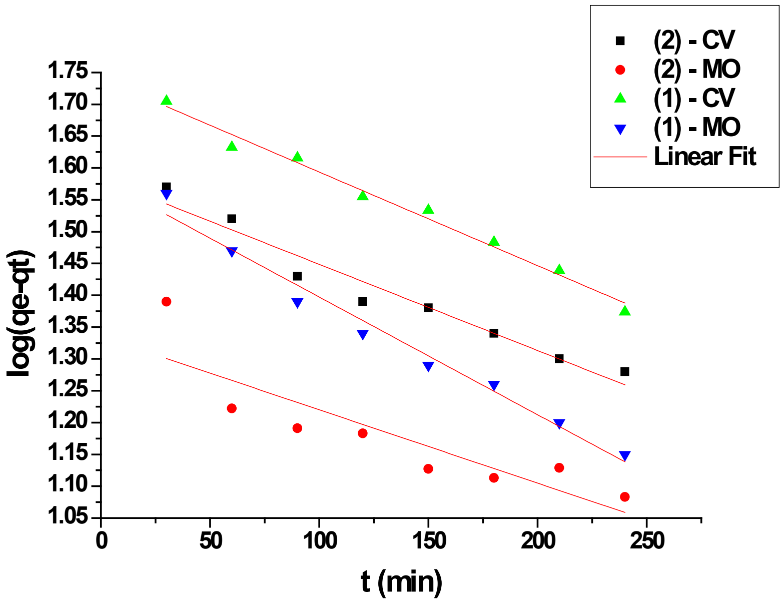

Supplementary Figure S7. Pseudo-first-order rate plot for the adsorption of CV and MO dyes by (1) and (2). For 100 ppm dye, adsorbent mass 0.05 g, volume solution 50 mL at 25 °C .

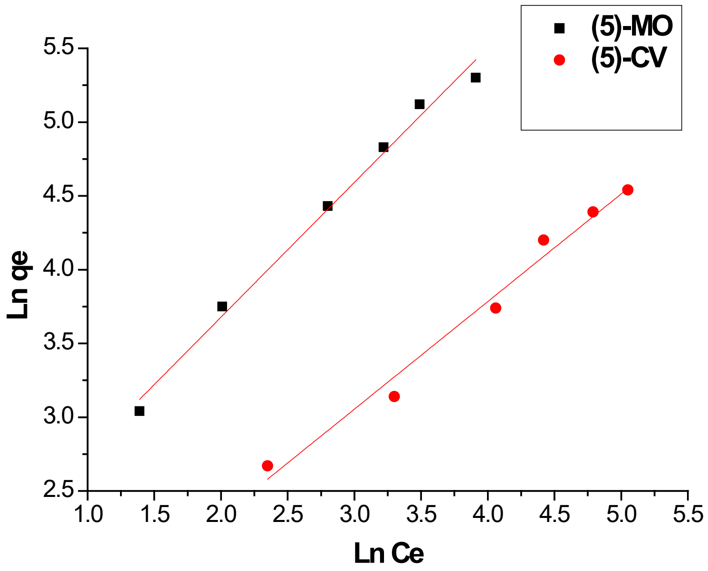

Supplementary Figure S8. Freundlich isotherm linear model for uptake of MO and CV dye by nanocomposites (5)

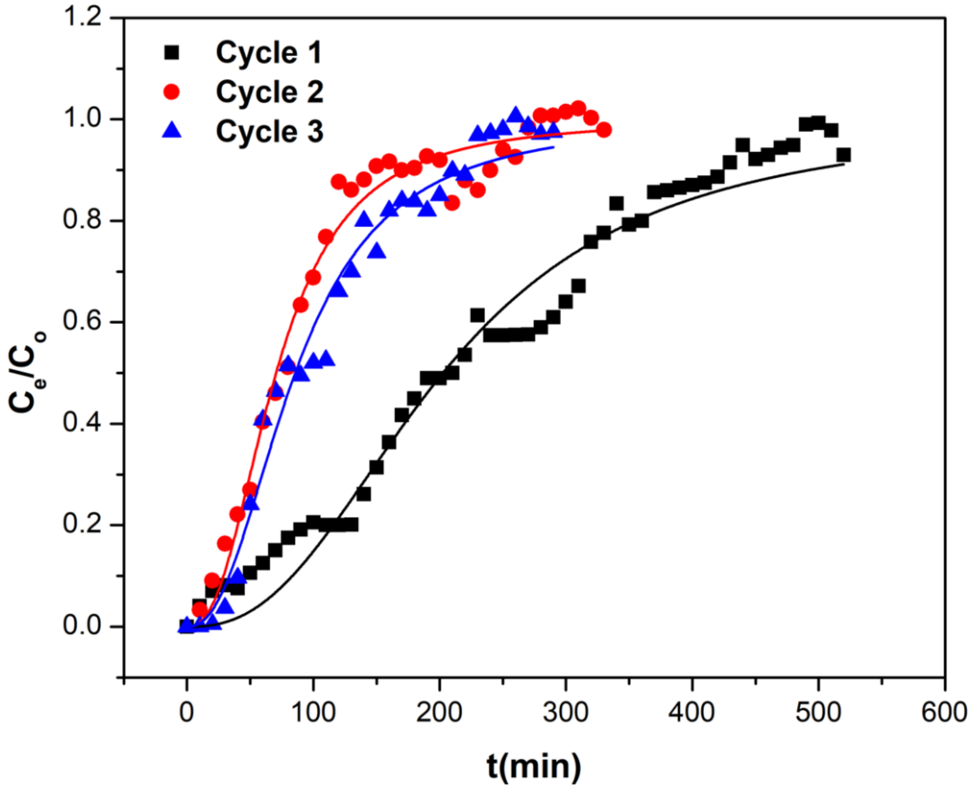

Supplementary Figure S9. Nonlinear fit of Yan et al. model for adsorption of CV by (1). (0.5 g of (1) packed in column, 100 ppm of CV dye, 1 mL/min flow rate)

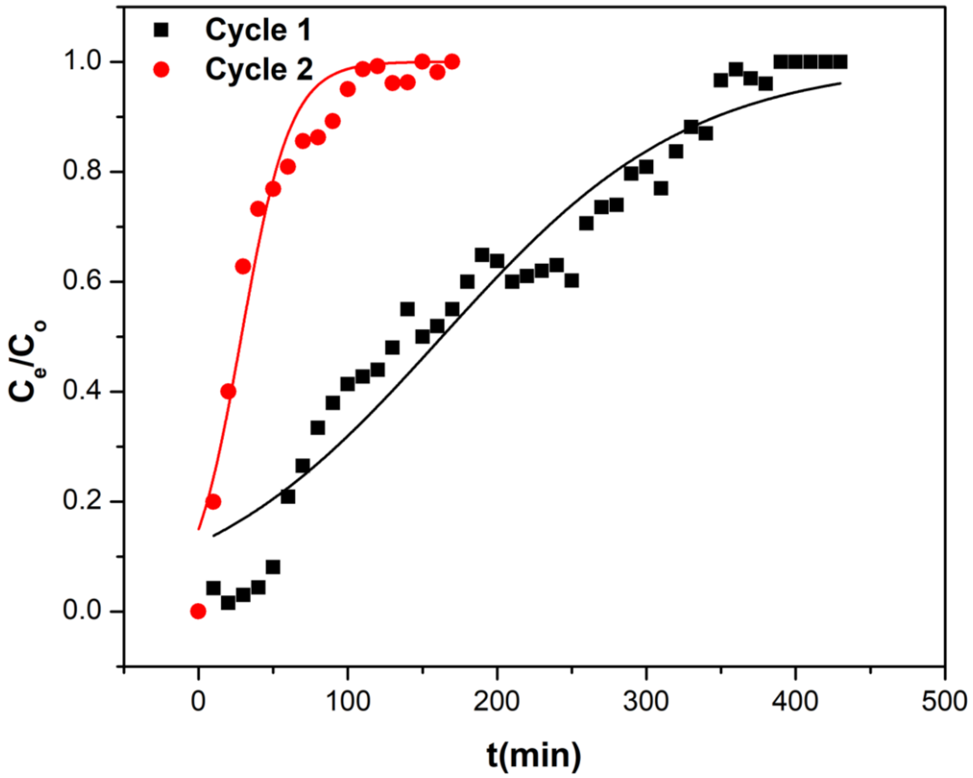

Supplementary Figure S10. Nonlinear fit of Thomas model for adsorption of MO by (1). (0.5 g of (1) packed in column, 100 ppm of MO dye, 1 mL/min flow rate)
